# Screening Cancer Immunotherapy: When Engineering Approaches Meet Artificial Intelligence

**DOI:** 10.1002/advs.202001447

**Published:** 2020-08-13

**Authors:** Xingwu Zhou, Moyuan Qu, Peyton Tebon, Xing Jiang, Canran Wang, Yumeng Xue, Jixiang Zhu, Shiming Zhang, Rahmi Oklu, Shiladitya Sengupta, Wujin Sun, Ali Khademhosseini

**Affiliations:** ^1^ Department of Bioengineering University of California, Los Angeles Los Angeles CA 90095 USA; ^2^ Center for Minimally Invasive Therapeutics California NanoSystems Institute University of California, Los Angeles Los Angeles CA 90095 USA; ^3^ Department of Chemical and Biomolecular Engineering Henry Samueli School of Engineering and Applied Sciences University of California, Los Angeles Los Angeles CA 90095 USA; ^4^ State Key Laboratory of Oral Diseases National Clinical Research Center for Oral Diseases West China Hospital of Stomatology Sichuan University Chengdu 610041 China; ^5^ School of Nursing Nanjing University of Chinese Medicine Nanjing 210023 China; ^6^ Department of Biomedical Engineering School of Basic Medical Sciences Guangzhou Medical University Guangzhou 511436 China; ^7^ Minimally Invasive Therapeutics Laboratory Division of Vascular and Interventional Radiology Mayo Clinic Phoenix AZ 85054 USA; ^8^ Harvard–Massachusetts Institute of Technology Division of Health Sciences and Technology Harvard Medical School Boston MA 02115 USA; ^9^ Jonsson Comprehensive Cancer Center University of California, Los Angeles Los Angeles CA 90095 USA; ^10^ Department of Radiology David Geffen School of Medicine University of California, Los Angeles Los Angeles CA 90095 USA; ^11^ Terasaki Institute for Biomedical Innovation Los Angeles CA 90064 USA

**Keywords:** artificial intelligence, cancer immunotherapy, drug screening, high‐throughput screening, tissue engineering

## Abstract

Immunotherapy is a class of promising anticancer treatments that has recently gained attention due to surging numbers of FDA approvals and extensive preclinical studies demonstrating efficacy. Nevertheless, further clinical implementation has been limited by high variability in patient response to different immunotherapeutic agents. These treatments currently do not have reliable predictors of efficacy and may lead to side effects. The future development of additional immunotherapy options and the prediction of patient‐specific response to treatment require advanced screening platforms associated with accurate and rapid data interpretation. Advanced engineering approaches ranging from sequencing and gene editing, to tumor organoids engineering, bioprinted tissues, and organs‐on‐a‐chip systems facilitate the screening of cancer immunotherapies by recreating the intrinsic and extrinsic features of a tumor and its microenvironment. High‐throughput platform development and progress in artificial intelligence can also improve the efficiency and accuracy of screening methods. Here, these engineering approaches in screening cancer immunotherapies are highlighted, and a discussion of the future perspectives and challenges associated with these emerging fields to further advance the clinical use of state‐of‐the‐art cancer immunotherapies are provided.

## Graphical abstract

Advanced engineering approaches and progress in artificial intelligence can contribute to the development of cancer immunotherapy by uncovering alternative treatment targets and identifying hallmarks of treatment response, efficacy, and side effects. Screening approaches, including sequencing, gene editing, tumor organoids engineering, bioprinting, and organs‐on‐a‐chip, combined with high‐throughput screening and artificial intelligence are promising in accelerating broader application of cancer immunotherapy for a larger population.

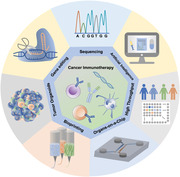

## 1 Introduction

The development of immunotherapies has revolutionized cancer management beyond the traditional treatment modalities including surgery, chemotherapy, and radiotherapy.^[^ ^1^, ^2^ ^]^ It has also yielded unprecedented clinical efficacy in treating some aggressive cancer types such as advanced melanoma and nonsmall‐cell lung cancer (NSCLC).^[^ ^3^, ^4^, ^5^ ^]^ Significant effort has been devoted to developing immunotherapies that are capable of boosting anticancer efficacy.^[^ ^6^ ^]^ In this regard, screening and identifying new targets that are related to the development of resistance or sensitization of tumors to immune responses could benefit the development of new immunotherapeutic strategies.^[^ ^7^ ^]^ From another perspective, the efficacy of cancer immunotherapy is largely dependent on the physiology of individual patient; across therapies and cancer types, ≈80% of patients are nonresponders or show severe side effects.^[^ ^8^ ^]^ Therefore, delivery of cancer immunotherapies has been identified as a key challenge in broadening its applicability, improving its efficacy, and reducing adverse effects; topics that have been recently reviewed from the perspective of biomaterials development^[^ ^9^ ^]^ and nanotechnology.^[^ ^10^, ^11^ ^]^ However, apart from challenging delivery, different preclinical and clinical studies have also suggested that the efficacy of immunotherapy could depend on patient‐specific mutagenesis, neoantigen load, and expression of certain biomarkers within the immune microenvironment,^[^ ^12^, ^13^, ^14^ ^]^ which makes predictive screening of immunotherapy on an individual patient basis essential as well. To achieve the goals of expanding the resources accessible for immunotherapy discovery and patient‐specific screening, platforms for the assessment of cancer immunotherapies at all levels (in vitro, ex vivo, in vivo) are indispensable.^[^ ^15^ ^]^

Advances in various engineering disciplines could help develop alternative cancer immunotherapy strategies to compensate for the unpredictable efficacy of current treatment. For instance, surface proteins expressed by cancer cells, such as PD‐L1, that help evade immune surveillance have been targeted with immunotherapeutic agents.^[^ ^16^ ^]^ However, many more genes that have similar functions may be alternative targets for development of new immunotherapeutic agents with high potency and efficiency. Another example is the engineering of tumor‐infiltrating lymphocytes (TILs) in which TCR genes can be introduced to generate tumor‐reactive T cells.^[^ ^17^ ^]^ Even though some genes shared by larger patient groups, such as NY‐ESO‐1, have been targeted, they are only expressed in a limited set of cancer types.^[^ ^18^ ^]^ Novel TCR gene screening shared by larger populations of patients with diverse malignancies could be of clinical significance. The advancement of precision medicine in disease management will also drive the need for developing individualized therapies with high precision to increase the number of responders treated with cancer vaccines and patient‐derived cell‐based immunotherapies.^[^ ^19^, ^20^, ^21^, ^22^ ^]^ Screening approaches using sequencing can help analyze patient tumor biopsies and healthy tissue to identify tumor‐specific mutations. These abnormalities can then be used to predict the binding affinity of mutated proteins to MHC molecules through computational approaches, and assist in the selection of optimal vaccine compositions for each individual.^[^ ^23^ ^]^ Time is also a primary concern when analyzing samples as it is desirable to minimize the time between the acquisition of the tumor biopsy and the administration of therapy. One study found that the median time from identifying appropriate cancer vaccine targets to administering treatment was 18 weeks, and patients suffering from advanced cancers may need expedited approaches.^[^ ^24^ ^]^ Therefore, it is expected that robust screening platforms enabled by a diverse set of engineering approaches will have a significant role in accelerating this process and advancing the broader applicability of cancer immunotherapy.

While the development of the screening platforms poses one challenge, clinically useful techniques must be scalable and facilitate processes that receive highly variable samples in order to enhance data reliability and normalize trials of treatment options.^[^ ^25^ ^]^ More importantly, certain recurrent patterns can only be identified and corroborated when sample diversity is sufficient and sample size is large. Due to the need for enormous quantities of data, analysis becomes a nontrivial issue that is too extensive for manual processing. Technology such as artificial intelligence (AI) could potentially be applied to analyze these large datasets and produce meaningful outputs.^[^ ^26^ ^]^ The overarching goal would be to distinguish tumor types or individual patients that are likely to yield responsive outcomes or show severe side effects with high fidelity and accuracy. The aggregation of data across multiple screening methods into a singular dataset can serve as a reference that catalogs the presence of biomarkers, outcomes of clinical tests, sequencing results, and patient responses to administered immunotherapies. The creation of datasets incorporating this information, expanding beyond the boundaries of current databases like The Cancer Genome Atlas (TCGA), will be essential in creating tools to personalize immunotherapy in clinical practices. Advanced screening models and data analytical techniques have great potential to be used preclinically and clinically to reduce treatment cost and improve patient response to cancer immunotherapies.^[^ ^27^, ^28^ ^]^

**Figure 1:**
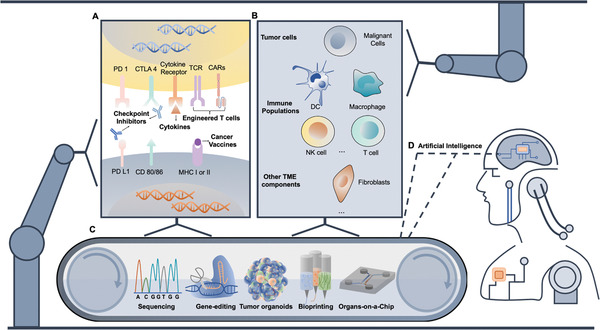
Engineering approaches with artificial intelligence for screening cancer immunotherapies. A) Cancer immunotherapies include cytokines, checkpoint inhibitors, adoptive cell transfer (ACT), and cancer vaccines targeting either tumor cells or dendritic cells and T cells. B) Diverse players involved in cancer immunotherapy include tumor cells, immune cells, stromal cells, and other tumor microenvironmental factors. C) Screening pipelines facilitated by sequencing, gene editing, tumor organoids engineering, bioprinting, and organs‐on‐a‐chip technologies can examine both intrinsic genetic features and recapitulate extrinsic factors. D) Those approaches can be scaled up aiming for high‐throughput screening and the resulting data can be processed by AI for rapid and accurate interpretations. Screening cycle enabled by various engineering approaches and AI is expected to accelerate the advancement of novel immunotherapy discovery and development.

In this review, we first discuss the indispensable need for screening four major classes of cancer immunotherapies (**Figure** **1**A). We then highlight recent research efforts using state‐of‐the‐art engineering approaches to build screening models aiming to improve the preclinical models used in drug development. Our focus ranges from leveraging intrinsic genetic factors, recapitulating extrinsic tumor microenvironment (TME) factors (Figure 1B), and reconfiguring data output for screening purposes based on sequencing, gene editing, tumor organoids engineering, bioprinting, and organs‐on‐a‐chip technologies (Figure 1C). We also present combinatorial designs with consideration toward establishing the next generation of screening platforms. As these systems are scaled into high‐throughput approaches, AI could facilitate the advancement in the applications of immunotherapies (Figure 1D). Finally, we discuss perspectives and mounting challenges in applying screening technologies for novel cancer immunotherapies such as combination and personalized therapies.

## 2 Classes of Cancer Immunotherapies

In this section, we briefly discuss cancer immunotherapies in the following categories: checkpoint inhibitors, cytokines, adoptive cell transfer (ACT), and cancer vaccines^[^ ^3^, ^29^, ^30^ ^]^—the most commonly used in the literature discussed below (**Table** **1**). The mechanism, significance, and corresponding challenges and limitations of each therapy are addressed in the context of their relevance to advanced screening approaches.

**Table 1:** Characteristics of different engineering approaches for cancer immunotherapies

Engineering approaches	Categories of cancer immunotherapy	Advantages	Limitations
Sequencing	Checkpoint inhibitors^[^ ^67^, ^68^, ^69^ ^]^ ACT^[^ ^70^ ^]^ Cancer vaccines^[^ ^71^ ^]^	Compatible with diverse cell types and patient samples Exhaustive characterization of genetic features High throughput	Costly and time consuming Data interpretation required Limited external control Limited prognostic value
Gene editing	Checkpoint inhibitors^[^ ^10^ ^]^ ACT^[^ ^72^, ^73^, ^74^ ^]^	Genome‐wide screening Controllable and designable Novel immunotherapy discovery High throughput	Low transduction efficiency Off‐target and false‐positive interpretation Costly and time consuming
Tumor organoid engineering	Checkpoint inhibitors^[^ ^75^, ^76^ ^]^ Cytokines^[^ ^75^ ^]^ ACT^[^ ^77^, ^78^ ^]^ Cancer vaccines^[^ ^77^ ^]^	Functional 3D model Immune population presence Ex vivo patient‐specific model buildup Capable of high throughput	May be difficult to fabricate Sample sourcing Long timeline for clinical decisions Long‐term TME diversity maintenance
Bioprinting	Cytokines^[^ ^79^, ^80^ ^]^	3D model with high spatial intricacy and resolution Recapture specific features and stages (such as lymphatic vessels, vasculature, and metastasis) Lowered batch‐to batch variability	Complex printing processes needed to maximize cell compatibility and fidelity Complexity of printing multiple cell types Mostly cell lines, not patient specific Difficult data collection Scalability challenges
Organs‐on‐a‐chip	Checkpoint inhibitors,^[^ ^81^, ^82^ ^]^ Cytokines^[^ ^81^ ^]^ ACT^[^ ^83^, ^84^, ^85^ ^]^ Cancer vaccines^[^ ^86^ ^]^	Microenvironmental control Physiological resemblance Built‐in readouts Recreate different features and stages (such as therapeutics transport)	Cellular fidelity under cell manipulation Mostly cell lines, not patient specific Scalability challenges

### 2.1 Checkpoint Inhibitors

Checkpoint inhibitors are the most extensively studied class of cancer immunotherapies with the most FDA approvals indicated for treatment of a variety of cancers.^[^ ^31^ ^]^ The existence of immune checkpoints are physiologically necessary as they balance immune activation and suppression to prevent the attack of healthy tissues and the occurrence of inflammation.^[^ ^32^ ^]^ Despite their essential roles in mediating autoimmunity, tumors also take advantage of these pathways to evade immune clearance. While several checkpoints have been identified, the interaction of PD‐1 with PD‐L1 and CD80/86 with CTLA4 are the most commonly targeted with therapies.^[^ ^33^ ^]^ Though expression by a variety of immune cells, mature effector T cells expressing PD‐1 can be deactivated by interactions with PD‐L1 expressed on the surface of tumor cells.^[^ ^34^ ^]^ CTLA4 operates in a similar fashion as it is a co‐inhibitory molecule that downregulates T cell activity through interactions with CD80 or CD86.^[^ ^35^, ^36^ ^]^ Checkpoint inhibitors are designed to block these pathways to prevent active T cells from binding the inhibitory ligands, thus improving their recognition, amplification, and killing capacity toward cancer cells. However, responsiveness toward checkpoint inhibitors varies significantly among cancer types, diseases stages, and patients,^[^ ^37^ ^]^ which necessitates screening for the purpose of response prediction. For instance, analysis of certain biomarker expression such as PD‐L1 on cancer cells was used to identify patient susceptibility to PD‐1 blockade therapies.^[^ ^38^ ^]^ In addition, the loss of immunogenic mutations or reduced expression of genes relevant to cancer immunotherapy under strong immune selective pressure can reshape the genetic landscape of cancers to limit sustained response to checkpoint inhibitors.^[^ ^39^ ^]^ Either tumor‐intrinsic or microenvironmental factors that dynamically shape immune interactions can be potentially recaptured by advanced screening strategies.

### 2.2 Cytokines

Cytokines were the earliest approved cancer immunotherapies and differ from checkpoint blockade in that they directly modulate and enhance the activity of immune cells.^[^ ^40^ ^]^ IL‐2, which gained FDA approval for treating metastatic melanoma and renal cell carcinoma in the 1990s,^[^ ^41^ ^]^ is one of the most common treatments in this class. Cytokines can be classified as interferons (such as INF‐*α* and INF‐*γ*), interleukins (such as IL‐2 and IL‐15), and colony‐stimulating factors (such as granulocyte‐macrophage‐CSF (GM‐CSF)),^[^ ^42^ ^]^ in which different subsets of immune cells or immune pathways can be selectively activated. Apart from extensively investigated cytokines, small molecules that induce cytokine production are also being studied including TGF‐*β* receptor type 1 inhibitors,^[^ ^43^ ^]^ TLR7/TLR8 agonists,^[^ ^44^ ^]^ and stimulator of interferon genes (STING) agonists.^[^ ^45^ ^]^ Nevertheless, cytokine‐based therapies are often associated with high doses and their action on regulatory T cells often causes severe side effects such as cytokine release syndrome and autoimmunity.^[^ ^46^ ^]^ Currently, it is not clear which biomarkers or genetic features are relevant for the identification of patients that can benefit long‐term from cytokine‐based immunotherapies. To address this issue, screening systems have been developed to evaluate safety and efficacy of therapies through the analysis of extrinsic factors such as T cell activation, survival, and proliferation or tumor cell death. Alternatively, intrinsic biomarkers associated with patient response could be identified through novel screening approaches.

### 2.3 ACT

ACT represents a “living” treatment that utilizes modified immune cells with direct anticancer activity harvested from the cancer‐bearing host.^[^ ^47^ ^]^ From the first successful demonstration of administrating TILs to patients with metastatic melanoma in 1988,^[^ ^48^ ^]^ the capability to reproducibly culture TILs with specific antitumor efficacy appears to be limited in melanoma.^[^ ^47^ ^]^ To expand the application of ACT to more cancer types, T cells can be genetically modified to enhance their specificity and efficacy against a tumor before being reinfused into the patient. Chimeric antigen receptor T cells (CAR T) and T cell receptor T cells (TCR T) are two major approaches in this category.^[^ ^49^, ^50^ ^]^ CAR T was initially designed to recognize CD19, a marker commonly expressed on B cell leukemias and lymphomas.^[^ ^51^ ^]^ Two of these genetically engineered cells (axicabtagene ciloleucel and tisagenlecleucel for lymphoma) have received FDA approval and are in clinical use. The success of early cell therapies has encouraged extensive efforts to design the next‐generation of CAR T with different targets.^[^ ^52^, ^53^, ^54^ ^]^ TCR T cells recognize tumor associated antigens presented by major histocompatibility complexes (MHCs) to target patient‐specific neoantigens originating from genetic mutations. The common challenges inhibiting the clinical use of engineered T cell therapies are the rapid identification of functional targets,^[^ ^55^ ^]^ the potential for cytokine release syndrome,^[^ ^56^ ^]^ and the low efficacy against solid tumors.^[^ ^57^ ^]^ Each of these challenges can benefit from advanced screening approaches for selecting specific antigens, evaluating cytokine release thresholds, and predicting efficacy in solid tumors.

### 2.4 Cancer Vaccines

Cancer vaccines are designed to elicit specific and potent immune responses toward cancerous tissues and mainly consist of four classes: tumor lysate, dendritic cells, nucleic acids, and neoantigens.^[^ ^58^, ^59^ ^]^ Whole tumor lysate vaccines include a more comprehensive array of immunogenic epitopes that can be used by dendritic cells to further propagate an immune response; however, clinical approval generally falters due to insufficient efficacy.^[^ ^60^, ^61^ ^]^ Dendritic cells can be pulsed by exposure to specific antigens in vitro to present tumor‐associated antigens that directly activate T cells to attack tumors. One dendritic cell vaccine called Sipuleucel‐T was approved by the FDA for the treatment of prostate cancer in 2010.^[^ ^62^ ^]^ In spite of excellent safety, low efficacy in inducing a sufficient immune response hinders its broader development and application. One proposed mechanism of improving efficacy is to identify and screen dendritic cell subtypes with high expression of targeted antigens.^[^ ^63^ ^]^ DNA or RNA‐based cancer vaccines display promising results by inducing antigen expression by antigen presenting cells (APCs) in vivo with high potency and relatively low cost.^[^ ^64^ ^]^ Apart from the delivery challenges, the selection and prediction of potent sequences that can induce strong and prolonged antigen presentation can be facilitated by screening strategies. Last, the immune system only targets neoantigens coming from tumor‐specific mutations that are exclusively present on cancer cells and thus prevent off‐target side effects.^[^ ^65^ ^]^ The identification of neoantigens for different cancer types or specific patients can be meaningful for the development of treatments effective against both heterogenous cancers and unique mutations.^[^ ^23^, ^66^ ^]^ Therefore, we mainly discuss screening approaches that can facilitate effective antigen selection and epitope prediction, as well as models showing how tumor cells, immune cells, and cancer vaccines interact with each other to elucidate treatment efficacy.

## 3 Engineering Approaches for Screening Cancer Immunotherapy

Immunotherapy screening methods are fundamentally complicated by the indirect action of the therapeutics. Unlike chemotherapeutics that have a direct mechanism of action against cancer cells, immunotherapies work to modify the patient's immune response to combat malignant cells.^[^ ^3^ ^]^ Therefore, models of cancer immunotherapy must incorporate additional components of the immune system in a physiologically relevant manner. Second, the diverse, heterogeneous phenotypes and genotypes of both cancer and immune cells dictate the discovery of new treatment targets and responses to treatment.^[^ ^15^, ^87^ ^]^ Developing models with a comprehensive representation of these aspects of the cancer–immune relationship is crucial for creating more effective screening protocols; however, achieving these two features in a single model is not trivial.^[^ ^88^ ^]^ Progress in biotechnology can therefore broaden the availability of treatment options, predict responses rates, and improve efficacy in the field of cancer immunotherapy by establishing screening models.^[^ ^9^, ^19^, ^23^ ^]^ Intrinsically, expression of certain genes or activation of specific pathways within cancer cells contribute to large variations in the efficacy of cancer immunotherapies.^[^ ^89^ ^]^ For instance, immunotherapy targets PD‐L1 and PD‐1 expressed on cancer cells and immune cells, and clinical responses of these therapies largely depend on PD‐L1 expression on TILs.^[^ ^90^ ^]^ Engineering approaches that can examine information at genomic and transcriptomic levels can be essential to facilitate screening for effective treatment.^[^ ^71^, ^91^ ^]^ Development of clustered regularly interspaced short palindromic repeats (CRISPR) systems will facilitate genome scale editing with a high level of versatility across a diverse group of cell types. Pooled screening models produced by CRISPR can be used for identification of targets capable of either boosting sensitivity or reversing resistance to immunotherapy.^[^ ^10^ ^]^ Extrinsically, complexity in the TME includes dynamic interactions among tumor cells, multiple subtypes of immune cells, and administered immunotherapeutic agents, all of which crucially regulate antitumor immune response.^[^ ^91^ ^]^ Technologies to develop tumor organoids recapitulating such complexity in vitro could function as miniaturized predictive screening models.^[^ ^92^ ^]^ Furthermore, bioprinting and organs‐on‐a‐chip technology can artificially reproduce temporal and spatial features of tumor–immune system and generate physiologically relevant models.^[^ ^93^, ^94^, ^95^ ^]^ Their versatility in biochemical and biophysical control can be advantageous for screening cancer immunotherapy.

### 3.1 Sequencing

Heterogeneity among cancer cells and T cells, as well as their interplay with the TME give rise to variable responses among patients and cancer types toward immunotherapies. Both intertumoral (among different patients) and intratumoral (within the same tumor) heterogeneity contribute to challenges in predicting how certain immunotherapeutic will interfere with a tumor.^[^ ^96^, ^97^, ^98^ ^]^ The development of next‐generation sequencing (NGS) techniques has revolutionized patient characterization, particularly in the context of patient screening. Cancer cells, stromal cells, and immune cells within the TME can be sequenced to reveal the hallmarks for treatment efficacy.^[^ ^99^ ^]^ The various methodologies of NGS have been comprehensively reviewed elsewhere.^[^ ^100^ ^]^ Advances in NGS have enabled exhaustive examinations of the genome and transcriptome by producing valuable datasets, which can be used to elucidate drivers of response. Whole genome sequencing (WGS) provides the most comprehensive information regarding a genome, however, cost is a limiting factor at the resolution needed to study both intertumoral and intratumoral heterogeneity essential for immunotherapy screening. In addition, a large proportion of information from WGS may not provide insightful input.^[^ ^101^ ^]^ Therefore, whole exome sequencing (WES) has been developed to only sequence genetic materials encoding for proteins. Due to the narrowed focus of the sequencing, more samples can be sequenced with higher efficiency to enhance both the breadth and depth of the intended studies. Other techniques in the category of NGS on the DNA level include Chip‐seq for probing DNA that can interact with proteins,^[^ ^102^ ^]^ ATAC‐seq for examining DNA unprotected by proteins, and methyl‐seq for capturing methylated DNA.^[^ ^100^ ^]^ All of which can be used to screen genes playing specific or unique roles in immunotherapy response. However, resistance or response prediction cannot be fully explained by genomic analysis and some recurrent features within the transcriptome may also contain useful information for predictive and prognostic purposes.^[^ ^103^ ^]^ Toward this goal, NGS enabled transcriptome profiling by using RNA‐sequencing (RNA‐seq) to decipher messenger RNA (mRNA) transcripts with high precision.^[^ ^104^ ^]^ Furthermore, combined assays sequencing both the genome and transcriptome of tumor tissue aim to elucidate response rates as a function of mechanism‐based variations while also looking to discover novel targets for additional therapies. Here, we present some research studies leveraging NGS for immunotherapy screening.

To better understand the mechanistic causes of variable response to checkpoint inhibitors and identify certain biomarkers for prognostic purposes, Van Allen et al. aimed to correlate individual response rate to anti‐CTLA4 with tumor mutational load, neoantigens, and cytolytic markers by performing both WES and RNA‐seq derived from 110 patients with metastatic melanoma.^[^ ^67^ ^]^ High correlation between mutational load and clinical benefit was observed for both DNA and RNA sequencing. By leveraging granzyme A and perforin as indicators for cytolytic activity of immune cells,^[^ ^39^ ^]^ transcriptome data from the TME correlated enrichment of these two genes in patients showing clinical benefit. Analysis at both the genomic and transcriptomic levels combined with clinical benefit correlation revealed that mutational load and immune microenvironmental cytolytic activity were predictive parameters for anti‐CTLA4 treatment, however, the limited number of patients in the study prevented the identification of reliable specific neoantigens as predictors of checkpoint inhibitor efficacy.^[^ ^65^ ^]^ Apart from tumor mutational burden (TMB) as a proven prognostic and predictive marker in cancer immunotherapies,^[^ ^97^ ^]^ there is also building evidence to suggest an uncertain relationship between TMB and anticancer response. For instance, one clinical trial tested first‐line anti‐PD1 in NSCLC and failed to prolong progression‐free survival in patients with >5% PD‐L1 expression.^[^ ^105^ ^]^ Therefore, holistic assessment of the TME, beyond neoantigen appearance and mutational burden, is required for different types of cancer.

**Figure 2:**
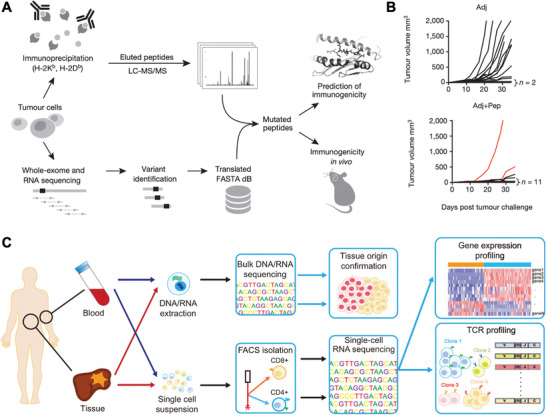
Sequencing for screening cancer immunotherapy. A) Schematic of combining sequencing and mass spectrometry to identify mutated peptides capable of being presented by MHC I. B) Tumor growth rate of mice immunized with either identified mutated peptide with adjuvants (Adj + Pep) or only adjuvant (Adj). Reproduced with permission.^[^ ^70^ ^]^ Copyright 2014, Nature Publishing Group. C) Workflow of applying sequencing for cancer immunotherapy screening from six treatment‐naïve hepatocellular carcinoma (HCC) patients based on exome‐ and RNA‐sequencing for identification of subsets of T cells. Reproduced with permission.^[^ ^71^ ^]^ Copyright 2017, Elsevier.

Aside from checkpoint inhibitors, NGS has also enabled the development and screening of cancer vaccines and ACT therapies. Certain somatic mutations can potentially evolve to be immunogenic if resulting mutant peptides are presented by MHC I as “nonself” antigens; if identified, they could be potentially formulated as cancer vaccines. Yadav et al. discovered immunogenic mutant peptides by combining exome and transcriptome sequencing with mass spectrometry (**Figure** **2**A). Integrated methods like this are powerful characterization tools for identifying mutated peptides presented by MHC I to facilitate personalized cancer vaccine development.^[^ ^71^ ^]^ The authors demonstrated feasibility of this approach in two murine tumor models, MC‐38 and TRAMP‐C1. After comprehensive combinatorial screening, only 3 candidates from MC‐38 had the ability to bind with MHC I and 0 candidate resulted from the TRAMP‐C1 tumor, which reflected low MHC I expression on TRAMP‐C1 cells.^[^ ^106^ ^]^ The identified mutant peptide vaccination successfully provided protective antitumor immunity to most animals after tumor challenge (Figure 2B). Beyond cancer cells, immune cells are the real “army” in the treatment, and function as a predictive marker of patient response. As shown in Figure 2C, over 5000 T cells derived from blood and tissues of patients with liver cancer underwent single‐cell RNA sequencing by Zheng et al. This led to the identification of 11 T cell subsets with corresponding signature genes.^[^ ^70^ ^]^ The ability to examine TCR sequences by RNA‐seq on a single cell basis is particularly important since TCRs are used to identify different T cell lineages, recognize the antigen presented by MHC, and control T cell activation.^[^ ^107^ ^]^ Identification of distinct features of T cell populations can therefore give insight and guidance into designing powerful ACT for cancer immunotherapy.

In short, NGS is a powerful tool for characterizing the intrinsic features of the various cellular components involved in cancer immunotherapy. Apart from its newly developed role in screening specific immunotherapies on certain patient populations, the already established sequencing databases are also meaningful for computer‐based analysis to derive clinically useful conclusions.

### 3.2 Gene Editing

The CRISPR system exists as an innate immune defense mechanism in bacteria and archaea using RNA to direct degradation of foreign DNA with high precision.^[^ ^108^ ^]^ Further development of this powerful tool has enabled site‐specific human genome editing with enhanced efficiency and simplicity.^[^ ^109^, ^110^ ^]^ More recently, it has been used to advance the systematic screening analysis of genetic functions,^[^ ^111^ ^]^ where large libraries of single guide RNAs (sgRNAs) encoded by lentiviral particles can produce a screening pool using cell lines or primary cells for screening cancer immunotherapies.^[^ ^74^ ^]^ Before the development of CRISPR, genetic diversity for screening was achieved either through random DNA mutations by mutagens or transcriptome silencing by RNA interference (RNAi), which were laborious and not exclusive to the genomic level.^[^ ^112^ ^]^ Cancer cells undergo constant neoplastic evolution with accumulated gene mutations^[^ ^113^ ^]^ that can induce either strong immune reactions due to the occurrence of neoantigens or, on the other hand, resistance to immunotherapies.^[^ ^114^ ^]^ In this regard, large variations in clinical response to therapies may result from diversified genetic profiles.^[^ ^67^, ^115^, ^116^, ^117^ ^]^ Benefiting from genetically diversified screening models facilitated by CRISPR technology, more targets can be identified for additional development of new therapeutics^[^ ^118^ ^]^ and the mechanisms leading to limited patient response can be better understood.^[^ ^10^ ^]^

**Figure 3:**
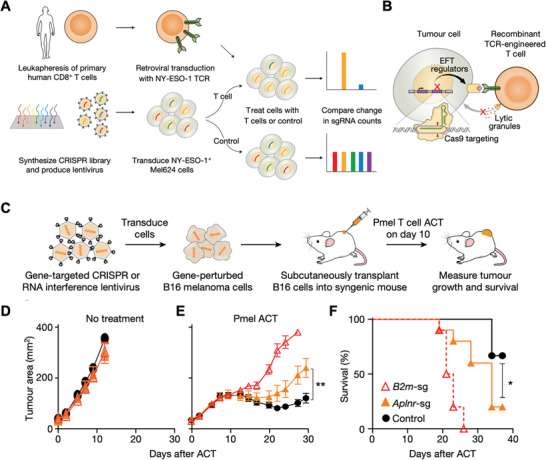
Gene editing for screening cancer immunotherapy. A) Workflow of genome‐wide loss‐of‐function screening via CRISPR for identification of genes causing resistance to T cell‐mediated cytolysis. B) Schematic of screening engineered T cells by CRISPR/Cas9 mediation. C) In vivo screening of ACT treatment on *β*‐2‐microglobilin (B2m) and apelin receptor (Aplnr) genes knockout tumor‐bearing immunocompetent mice. D,E) Tumor area growth with no treatment (*n* = 5) or treated by ACT of Pmel‐1 T cells (melanoma antigen gp100‐specific transgenic T cells). F) Overall survival of mice treated with Pmel‐1 T cells. (Red hollow triangle, orange triangle, and black circle in (D)–(F) indicate B2m‐sg (*n* = 10), Aplnr‐sg (*n* = 10), and control (*n* = 9) groups, respectively; all values are mean ± s.e.m. ^**^ *P* < 0.01 and ^*^ *P* < 0.05.) Reproduced with permission.^[^ ^72^ ^]^ Copyright 2017, Nature Publishing Group.

CRISPR‐created screening pools have been used in human cell lines to reveal gene targets involved in T cell‐based therapies. Patel et al. utilized genome‐scale editing to mimic loss‐of‐function mutations for a melanoma cell line by implementing CRISPR/Cas9 with ≈123 000 single‐guide RNAs. As shown in **Figure** **3**, the authors developed a “two cell type” (2CT)‐CRISPR assay in which Mel624 cells were transduced with a CRISPR knockout library and reacted with human T cells expressing a specific T cell receptor (TCR) for the NY‐ESO‐1 antigen. The screening assay was capable of studying how effector T cells respond to genetically manipulated tumor cells and it was demonstrated in vivo that multiple mutations in APLNR genes related to cellular signaling could sensitize the tumor toward ACT immunotherapy.^[^ ^72^ ^]^ In terms of checkpoint blockade therapies, low response rates suggest the need for the discovery of novel targets. Manguso et al. used CRISPR/Cas9 to create a pool of loss‐of‐function melanoma cell lines in vitro and then transplanted the whole library in vivo to screen immunotherapy‐related resistance and sensitivity. The B16 melanoma cell line was engineered to express Cas9 and was transduced with lentiviral vectors encoding 9872 sgRNAs. They validated the widely targeted molecules PD‐L1 and CD47 as contributors to resistance to immunotherapy and also identified multiple genes that can sensitize tumors to immunotherapy if lost, such as protein tyrosine phosphatase PTPN2.^[^ ^10^ ^]^ The screening strategy, by Pan et al., was to transduce genome‐wide gRNAs within Cas9‐expressing B16F10 cells, followed by coculturing with cytotoxic T cells and subsequent Illumina sequencing of gRNA representation. Through this process, the authors identified intrinsic genes in tumor cells as additional mechanisms that regulate killing activities of cytotoxic T cells through CRISPR‐Cas9 screens.^[^ ^73^ ^]^

Immortalized cell line‐based CRISPR screens may not fully resemble the signaling pathways and functions in humans, which could slow down the translational process of newly discovered targets into clinics. To compensate for this disadvantage, Shifrut et al. reported a CRISPR‐Cas9 screening platform in primary human T cells to enable the discovery of more relevant targets. The authors utilized pooled lentiviral sgRNA followed by electroporation of Cas9 protein to overcome the traditionally low transduction rate in primary cells. The pooled loss‐of‐function screening allowed the selection of T cells with enhanced in vitro anticancer efficacy and targets that could evade immunosuppression.^[^ ^74^ ^]^ Parnas et al. performed a genome‐wide CRISPR screen for primary dendritic cells isolated from Cas9‐expressing mice that were utilized to identify regulatory networks.^[^ ^119^ ^]^ Ex vivo or in vitro screens still face challenges such as the limited viability of primary cells and limited physiological relevance due to artificial manipulations. To overcome this challenge, LaFleur et al. reported an immune editing platform that allows direct gene perturbation in vivo while minimizing alterations to cell development and function as a result of ex vivo manipulation. The authors transduced bone marrow precursors with CRISPR‐Cas9 and enabled gene deletion in both innate and adaptive immune cell populations without altering cell states. The capability to edit additional subsets of immune cells, such as macrophages and dendritic cells, may facilitate the discovery of alternative immunotherapeutic targets beyond T cells.^[^ ^120^ ^]^

Gene editing technologies enabled by CRISPR allow the artificial creation of screening models with unprecedented precision and diversity. In tandem with NGS, these techniques can shed light on mechanisms and targets associated with sensitivity and resistance to immunotherapy.

### 3.3 Tumor Organoids

Advances in tumor organoid engineering have provided an alternative immunotherapy screening model that can recapitulate the TME and preserve innate immune–tumor interactions ex vivo in 3D.^[^ ^121^ ^]^ Additionally, many researchers are looking to overcome the challenges associated with predicting individual responses in terms of efficacy and safety by integrating patient‐derived tumor organoids (PDOs) into screening platforms.^[^ ^122^ ^]^ Conventionally used human cancer models are still limited to cancer cell lines, primary patient‐derived tumor xenografts (PDTXs), or genetically engineered mouse models (GEMMs) that often fail to satisfy the requirements of novel immunotherapy screening.^[^ ^123^ ^]^ Cancer cell lines derived from patient materials need extensive adaptation for in vitro culture. Clones capable of expanding limitlessly are rare and normally subject to drastic genetic changes, making them unrepresentative of the original tumor.^[^ ^124^ ^]^ The original genetic heterogeneity of the tumor is also lost, as mutated variants compete to expand ex vivo. PDTXs are expensive and time‐consuming, while tumorigenesis may evolve in a host species specific manner^[^ ^122^ ^]^ which may be unsuitable to the short timeframes needed for clinical scenarios. Facile strategies to produce organoids with the ability to recapitulate 3D interactions among the tumor, immune cells, and immunotherapeutic agents are candidates for screening.

Advances in the efficient culture of organoids derived from adult human stem cells have laid the foundation for PDOs culture and has been used in several studies focusing on breast cancer,^[^ ^125^ ^]^ prostate cancer,^[^ ^126^ ^]^ colorectal cancer (CRC),^[^ ^127^ ^]^ NSCLC,^[^ ^77^ ^]^ pancreatic cancer,^[^ ^128^ ^]^ and liver cancer.^[^ ^129^ ^]^ PDOs are expanded from the tumor of an individual patient with high success rates. Directly sourcing the cells from the patient tumor can preserve both the genetic and morphological features found in original tumors for establishment of a personalized screening platform.^[^ ^121^ ^]^ The inherent diversity within the tumor microenvironment including the stroma, vasculature, and immune cells makes it challenging to recreate directly, thus coculture can be a functional strategy to artificially combine different components. Dijkstra et al. recreated cancer‐T cell interactions by coculturing autologous tumor organoids and peripheral blood lymphocytes (PBLs) from the same patient in order to enrich tumor‐reactive T cells and assess corresponding killing. The authors demonstrated that this strategy can be successfully applied to epithelial cancer types, such as CRC and NSCLC, with T cell response specific to the corresponding tumor organoids. The tumor‐reactive T cells were expanded by an established protocol for ACT^[^ ^130^ ^]^ and efficient killing was only observed in malignant, not healthy, organoids. Furthermore, the presence of antibodies blocking MHC I and MHC II can inhibit the killing of tumor organoids. Minimally invasive acquisition of paired tumor and T cells by needle biopsies and peripheral blood allows the patient‐specific study of resistance and sensitivity to immunotherapy.^[^ ^77^ ^]^

**Figure 4:**
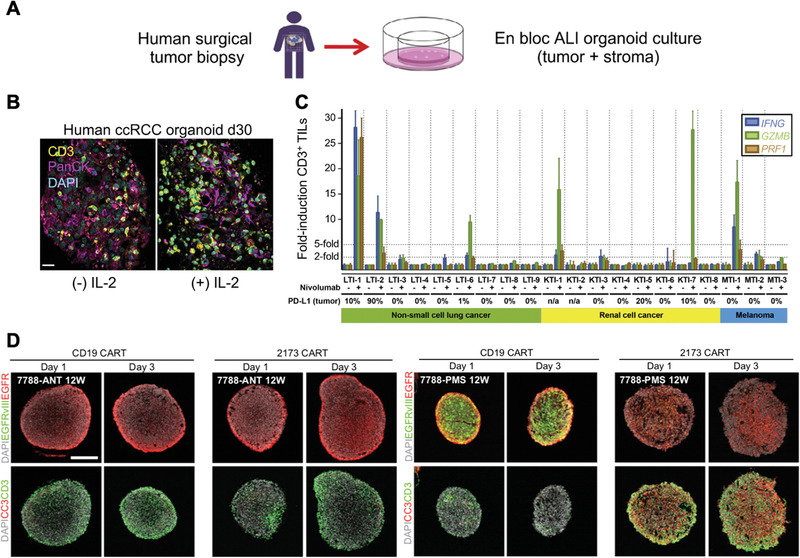
Tumor organoids engineering for screening cancer immunotherapies. A) Patient‐derived tumor organoid (PDO) fabrication via an air–liquid interface (ALI) method from human surgical tumor biopsies. B) Representative immunofluorescence staining of CD3^+^ tumor infiltrating lymphocytes (TILs) with or without IL‐2 treatment, scale bar = 20 µm. C) qRT‐PCR analysis of interferon‐gamma (IFNG), granzyme B (GZMB), and perforin‐1 (PRF1) of CD3+TILs from NSCLC, RCC, and melanoma PDOs after anti‐PD1 or control IgG4 treatment for 7 days. Reproduced with permission.^[^ ^75^ ^]^ Copyright 2018, Elsevier. D) Confocal images of 1 day and 3 day coculture of CAR T (either CD19 or 2173 BBz CAR T cells, 2173BBz CAR T cells target specifically EGFRvIII expressing cells) with glioblastoma organoids (GBOs) by immunostaining EGFR, EGFRvIII, Cleaved‐caspase‐3 (CC3), and CD3. Reproduced with permission.^[^ ^78^ ^]^ Copyright 2020, Elsevier.

Direct strategies to maintain the innate TME within organoids require novel methods of organoid fabrication. Neal et al. utilized an air–liquid interface (ALI) method to successfully build PDOs from 100 individual patient tumor biopsies generated from different organs (**Figure** **4**A) that recapitulate the immune TME. The ALI method is distinct in that it also maintains the stromal component with the PDOs. Diverse types of immune cell populations were detected within the PDOs including cytotoxic T cells, helper T cells, B cells, natural killer (NK) cells and tumor‐associated macrophages (TAMs). It was also found that administration of IL‐2 preserved the viability of TILs (Figure 4B). Apart from the platform itself, the authors also developed a downstream assay that determines the immune cell subsets conserved between tumors and PDOs. Last, they utilized immunotherapy‐responsive tumors to generate 20 PDOs and followed with anti‐PD1 treatment. Activation of T cells was observed and tumor killing activity was noticed in 6 PDOs by assessing interferon‐gamma (IFNG), granzyme B (GZMB), and perforin‐1 (PRF1) of CD3+TILs, data that is consistent with clinical trials for different cancer types (Figure 4C). The developed PDOs may facilitate predictive evaluation of responses to clinically applied immunotherapies including cytokines and checkpoint inhibitors on an individual basis.^[^ ^75^ ^]^ Another strategy applied by Votanopoulos et al. engineered immune‐enhanced patient tumor organoids (iPTOs) by dissociating surgically obtained tumor and lymph node biospecimens without sorting cells to preserve tumor heterogeneity. The incorporation of the lymph node better represented populations of APCs and allowed activation of T cells for killing activities. The authors also demonstrated high clinical correlation (85%) with response to checkpoint inhibitors.^[^ ^76^ ^]^ Organoids can be adapted for engineered T cell screening as well. Jacob et al. reported a strategy to biobank patient‐derived glioblastoma (GBM) organoids with high resemblance to parental tumors in terms of histological, cellular, genetic, and mutational features. They demonstrated the feasibility of screening personalized CAR T therapies using the organoids in which antigen‐specific recognition and killing were observed (Figure 4D). This 3D model can function as an effective platform to test and optimize CAR T treatment for solid tumor in vitro before conducting in vivo test or human trials.^[^ ^78^ ^]^ However, it should be noted that successful derivation of organoids depends on tissue acquisition, tissue quality, and corresponding tumor compositions and growth features. Low efficiency in establishing and propagating these cultures may fail to meet the demands of clinical use. In addition, even though preserved immune cells and microenvironmental characteristics can be detected initially, they can be lost and diluted over time as in vitro culture conditions favor clones optimized for tumor growth.

### 3.4 Bioprinting

**Figure 5:**
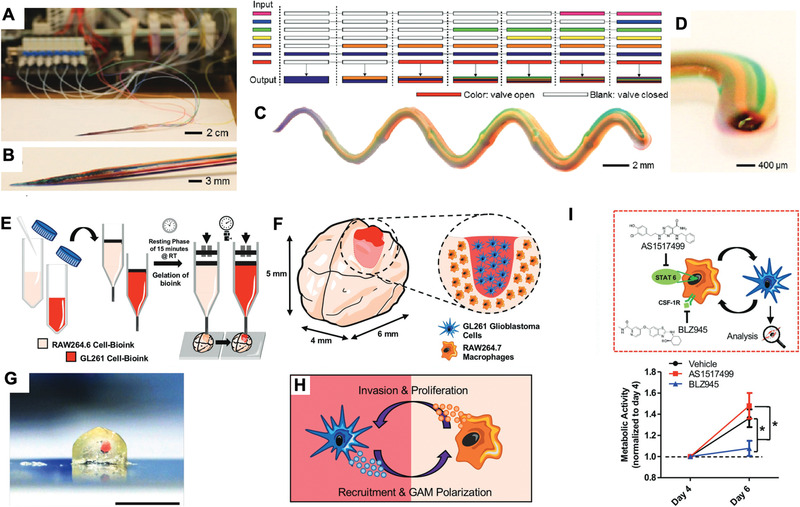
3D bioprinting for screening cancer immunotherapy. A,B) Photograph of a seven‐channel printhead of a continuous multimaterial extrusion bioprinter. C) Photograph of a printed microfiber with a seven‐bioink composition. D) Magnified photograph (side view) of the printed microfiber. Reproduced with permission.^[^ ^79^ ^]^ Copyright 2016, Wiley‐VCH Publications. E) Preparation of cell‐laden GelMA‐gelatin bioinks and the subsequent bioprinting process to create a 3D glioblastoma screening model (minibrains). F) Magnified schematic representation of bioprinted minibrains. G) Magnified cross‐section image of bioprinted minibrains with glioblastoma area highlighted in red, scale bar = 5 mm. H) Crosstalk between macrophages and glioblastoma cells. I) (Top) Schematic of immunomodulatory drugs AS1517499 and BLZ945 treatment targeting on macrophages for glioblastoma cell behaviors modulation. (Bottom) Measurement of metabolic activity of glioblastoma cells treated by AS1517499 and BLZ945 on day 4 and day 6, ^*^ *P* < 0.05. Reproduced with permission.^[^ ^131^ ^]^ Copyright 2019, Wiley‐VCH Publications.

Oversimplified in vitro tumor models with limited TME relevance may be remedied by the progress in bioprinting. The technology can precisely distribute and organize multiple biological components with high spatial resolution and uniformity, paving the way for high‐content models with low batch‐to‐batch variability (**Figure** **5**A–D).^[^ ^131^, ^132^, ^133^, ^134^ ^]^ Multiple bioprinting strategies exist and primarily vary in their mechanisms of bioink deposition. Major types include droplet‐based, extrusion‐based, and laser‐based printing,^[^ ^135^ ^]^ all of which have been used to facilitate in vitro cancer modeling.^[^ ^136^, ^137^ ^]^ Droplet‐based bioprinting utilizes thermal or acoustic energy to generate ink droplets, while extrusion‐based is driven by mechanical or pneumatic force to yield continuous fiber extrusion. In contrast, laser‐assisted bioprinting uses focused light to crosslink specific points within a biomaterial solution to generate 3D structures in a layer‐by‐layer fashion.^[^ ^138^ ^]^ The characteristics of different fabrication strategies could be tailored to fulfill different features of tumor, immune, and TME interactions. This can be done by designing bioink compositions with various compositions of extracellular matrix (ECM) proteins, an important feature of complex TME models. Diverse biomaterials, such as naturally derived or synthetic bioinks, generally have tunable biochemical and biophysical properties. For instance, matrix stiffness can be modulated by altering the composition and concentration of bioinks and postprinting processing. For the purposes of screening cancer immunotherapy, screening requirements can be met by a variety of advances in bioprinting. Matrix stiffness is of importance in tumor evolution^[^ ^139^ ^]^ and the immune cell niche can be significantly impacted by mechanical characteristics.^[^ ^140^ ^]^ Additionally, bioprinting is especially advantageous in creating intricately entangled passageways that closely mimic the physiological networks of vasculature and lymphatic pathways.^[^ ^141^, ^142^, ^143^, ^144^ ^]^ These microscale channels created by bioprinting can facilitate drug transport and nutrient exchange that are meaningful for screening cancer immunotherapy.

To study the crosstalk between tumor and immune cells in the presence of immunomodulatory drugs, Heinrich et al. 3D bioprinted a miniaturized brain that recaptured the interactions between GBM and TAMs in a biomimetic manner (Figure 5E–J).^[^ ^79^ ^]^ Transcriptomic analysis of the system showed that certain overexpressed genes in 3D bioprinted minibrains correspond to GBM patient clinical data. However, several discrepancies were found indicating further regulation by other TME components. The macrophages embedded within the miniaturized brain are recruited by glioblastoma cells and can be polarized into an M2 phenotype that favors tumor growth. They finally screened two immunomodulatory drugs: colony stimulating factor 1 receptor (CSF‐1r) inhibitor, BLZ945, and STAT6 inhibitor, AS1517499. Treatment with BLZ945 in the system yielded results in alignment with the reported in vivo study showing that BLZ945 can directly affect GBM growth by downregulating markers of M2 phenotype.^[^ ^145^ ^]^ 3D bioprinting is also suitable for generating spatially complicated structure to better mimic physiological interactions between different cell types. Kilian et al. utilized coextrusion bioprinting to study paracrine crosstalk study between MDA‐MB 231 breast cancer cells and macrophages.^[^ ^80^, ^146^ ^]^ Tumor cells were embedded within an alginate solution as the shell of the fiber and macrophages were loaded into a CaCl_2_ solution in the core to crosslink the viscous alginate with Ca^2+^ ions. The authors demonstrated a range of geometric shapes of microchannels can be achieved via bioprinting, which created different diffusional distances and spatial arrangement of cells mimicking normal physiological processes. They recapitulated both macrophage migration and inhibition of migration through drug administration. This method of extruding alginate fibers is compatible with high‐throughput screening for various drugs.^[^ ^146^ ^]^ The in vivo TME also relies on embedded microcirculation systems with perfusion and drainage networks coming from both blood and lymphatic vessels, key components in the transport and delivery of therapeutics, including cancer immunotherapeutic agents. Cao et al. established a 3D tumor model with a dynamic microenvironment achieved by the inclusion of a pair of perfusable blood vessels and one end‐blinded lymphatic vessel. The permeability of both vessels can be tuned to match their native properties by changing the composition of the bioinks to yield different diffusion profiles for biomolecules and small molecule drugs. Physiologically relevant circulatory platforms facilitate the study of drug kinetics and transportation, which undoubtedly play a role in the efficacy of cancer immunotherapies.^[^ ^147^ ^]^ However, this role is relatively undefined and is not mechanistically characterized.

Bioprinting is advantageous in establishing spatially intricate structures for screening that are physiologically essential and otherwise challenging to achieve. Although in principle bioprinting is capable of adding multiple cellular components with ease, inclusion of both spatial intricacy and cellular diversity simultaneously remains challenging and necessitates further development of bioprinting technologies.

### 3.5 Organs‐on‐a‐chip

Bridging between 2D cell culture and in vivo systems, organs‐on‐a‐chip platforms have the potential to rebuild key structural and functional components of organs with human cells as cell sources for extended analysis, manipulation, and screening.^[^ ^148^ ^]^ Organs‐on‐a‐chip platforms are developed around microfluidic systems, which enable external biochemical and biophysical control used to recreate physiological features of the TME.^[^ ^149^ ^]^ In addition, organs‐on‐a‐chip platforms can be easily integrated with add‐ons such as sensing and imaging to facilitate facile data collection for analysis. Furthermore, the use of patient‐derived cells facilitates standardized, economical, and personalized ex vivo screening models that can be built to enable the investigation of basic biological processes and the preclinical validation of drugs—essential tools for screening personalized and/or precision therapies.^[^ ^148^, ^150^ ^]^ Specific to cancer immunotherapies, the immune system plays an important role in the occurrence, development, and metastasis of cancers. Interactions between healthy and malignant tissues and cells can be recaptured through organ‐on‐a‐chip systems with the ability to monitor key parameters and cellular behaviors such as cytokine secretion, immune cell infiltration, and tumor viability and migration. In this context, cancer‐on‐chip systems with immune components have been established for therapeutic screening and investigation of the interactions between cancer cells and immune cells.^[^ ^150^, ^151^ ^]^ Here, we summarize recent progress in the development of organs‐on‐a‐chip systems for immunotherapy screening.

Organs‐on‐a‐chip systems can be fabricated with simple designs to provide powerful biological relevance to facilitate rapid screening on an individual patient basis. For screening the therapeutic efficacy of adoptive T cell therapies, solid tumors create a notoriously challenging environment for T cells function because of their dense structure and immunosuppressive biochemical cues. Park et al. reported a screening model based on an injection‐molded culture platform for 3D cytotoxicity assays (CACI‐IMPACT). The chip was designed to compartmentalize different cell types and ECM gels. By adding the components of ECM, which act as barriers to restrict the migration of cytotoxic lymphocytes, the chip system mimics the native TME and can be used to evaluate the killing capacity of engineered T cells.^[^ ^83^ ^]^ In addition to evaluating the killing ability, specificity is another important parameter to be tested in screening effective treatments. TCR T cells or CAR T cells are developed to recognize specific antigens presented by cancer cells. Screening is a crucial step in ensuring their specificity is selective for malignant tissue to prevent off‐target effects and autoimmunity. To satisfy this requirement, Segaliny et al. developed a droplet microfluidic system combined with tracking and sorting capabilities to screen antigen specific T cells and monitor, in real time, single TCR T cell activation by target tumor cells. The single cell screening was achieved by coencapsulating TCR T cells and antigen‐specific cancer cells within one droplet. The activation process was visualized by green fluorescent protein (GFP)‐reporter T cells where recognition of the tumor antigen induced GFP expression. After identifying and isolating the activated T cells, these cells underwent downstream sequencing and TCR chain screening was used to identify TCR sequences specific to antigens on a single cell level.^[^ ^84^ ^]^ Advanced technologies are easily incorporated with organs‐on‐a‐chip platforms for more sophisticated purposes. Ke et al. reported a chip equipped with titanium oxide phthalocyanine (TiOPc)‐based optoelectronic tweezers (OET) for real‐time examination of the dynamic interactions between immune cells and cancer cells. The chip can observe activity of NK cells and apoptotic features indicating dying over time. Using this method, the dynamic process of NK cell killing target cells was observed because of spatial control of cell–cell contact from the OET.^[^ ^85^ ^]^

### 3.6 Hybrid Design

**Figure 6:**
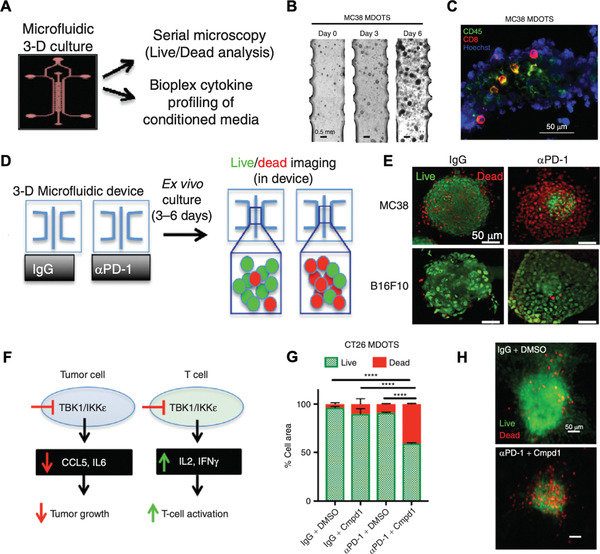
Organoids‐on‐a‐chip for screening cancer immunotherapy. A) Schematic design of a microfluidic chip system and downstream analysis with fluorescence imaging and cytokine secretion analysis for 3D cultured murine and patient‐derived organotypic tumor spheroids (MDOTS/PDOTS). B) Brightfield image of MC38 MDOTS on day 0, day 3, and day 6. C) Immunofluorescence staining of immune populations in MC38 MDOTS by anti‐CD45 and CD8 antibodies. D) Schematic of checkpoint inhibitor screening via chip system followed with live/dead imaging. E) Live (green)/dead (red) fluorescence image of MC38 and B16F10 MDOTS treated with anti‐PD1 or control IgG. F) Schematic of impact on cytokine secretion of tumor cells and immune cells by a TBK1/IKK*ε* inhibitor. G,H) Live (green)/dead (red) quantification and fluorescent image of CT26 MDOTs treated with or without anti‐PD1 and with or without TBK1/IKK*ε* inhibitor (Cmpd1), ^****^ *P* < 0.0001. Reproduced with permission.^[^ ^81^ ^]^ Copyright 2017, American Association for Cancer Research.

Each screening approach has unique advantages and disadvantages when surveying the immune–cancer interactions in the TME to aid novel immunotherapy discovery or efficacy and safety evaluations. Thus far, none of the approaches has had absolute success predicting efficacy and each is inadequate on its own. Therefore, researchers are beginning to combine multiple approaches synergistically to establish more powerful screening models. Though these studies are still nascent for immunotherapy applications, they do hold great potential. One common hybrid design is the organoids‐on‐a‐chip,^[^ ^152^ ^]^ which combines two distinguished approaches to increase the relevance for cancer immunotherapy screening.^[^ ^153^ ^]^ Patient‐derived tumor organoids recapture the functional and structural diversities corresponding to individual patients and cancer types, but still lack the microenvironmental controls and facile analytical methods. Organs‐on‐a‐chip technologies can easily incorporate microfluidic channels and integrate with sensing components to facilitate monitoring.^[^ ^154^, ^155^ ^]^ Varying the chip design to facilitate other unique features, such as cell separation, can be used to supplement downstream analysis such as sequencing and functional response screening.^[^ ^156^ ^]^ As displayed in **Figure** **6**, Jenkins et al. reported a 3D microfluidic device for short‐term culture of both murine and patient‐derived organotypic tumor spheroids (MDOTS/PDOTs) with preserved immune cell populations. Combining the advantages of preserved tumor–immune interactions and real‐time monitoring, checkpoint inhibitors and small molecule drugs can be delivered through microfluidic channels and both live/dead screening and cytokine profiling can be conducted in the device. They demonstrated ex vivo sensitivity and resistance to PD‐1 blockade therapy within a short period (3–6 days), showing potential to quickly evaluate immune checkpoint blockade (ICB)‐based therapies in a clinical setting. They also screened a small‐molecule inhibitor targeting the TBK1/IKK*ε* interaction aimed at sensitizing the organoids to checkpoint inhibitor treatment.^[^ ^81^ ^]^

Single cell or single cell type sequencing is of interest in screening cancer immunotherapies as the immune microenvironment is complicated with different subsets of cell populations, each with their own distinct functions.^[^ ^70^ ^]^ Just as microfluidic systems can be used to sort cells in preparation for sequencing, CRISPR‐editing can complement organoids use. Since the advent of the first CRISPR application in organoids for disease modeling in 2013,^[^ ^157^ ^]^ CRISPR has been applied for tumorigenesis modeling, genome‐wide organoid screening, and development of reporter organoids.^[^ ^158^, ^159^ ^]^ Gene‐editing can generate artificial organoids to exhibit distinct functions and properties that naturally derived organoids may fail to replicate ex vivo. Furthermore, combinations of bioprinting and organs‐on‐a‐chip techniques are gaining popularity.^[^ ^95^, ^160^ ^]^ Meng et al. 3D printed a metastatic model organ‐on‐a‐chip combining primary tumor, vasculature, and stromal elements with the ability to screen immunotoxins. The efficacy of the immunotoxins assessed with the bioprinted organ‐on‐a‐chip platform manifested more meaningful cellular responses for tested drugs.^[^ ^161^ ^]^ It is expected that future screening models for cancer immunotherapy will be enhanced by combining two or more approaches with both intrinsic and extrinsic features represented.

## 4 Artificial Intelligence in Screening Cancer Immunotherapy

To better fulfill the purpose of screening models as predictive or discovery platforms, clinically useful and commercially feasible engineering approaches must be compatible with high‐content or high‐throughput settings. Many of the platforms we have discussed are, in principle, amenable to scale up to enhance their efficacy, efficiency, and accuracy. Data acquisition and interpretation is challenging, however, significant advancements in AI can support the growing need for big data analytics. In this section, we discuss strategies for scaling up each engineering approach either alone or in combination and highlight research studies applying AI to complement high‐throughput screening models.

### 4.1 High‐Throughput Screening

Broad scale commercial and clinical use of screening methods requires high‐throughput systems capable of rapid sample testing and data analysis. Tumor organoids are naturally compatible with high‐throughput setups, but samples derived from patients vary in quantity and quality and pose challenges for this purpose. Phan et al. utilized the geometry of the commercial well plates to generate Matrigel‐embedded PDOs within rings along the rim of the well.^[^ ^162^ ^]^ The adopted strategy was simple in scalability and allowed the use of limited cell numbers from patient samples to be capable of performing hundreds of parallel independent screenings. One crucial feature the authors achieved was to gain screening results within one week after surgery, which is compatible with the clinical decision‐making process. Organs‐on‐a‐chip platforms or traditional well plate platforms can be designed for high‐throughput screening. Mo et al. developed a high‐throughput screening platform for small‐molecule immunomodulator discovery (HTiP) within a 384‐well plate. Tumor immune interactions were recapitulated by coculturing PBMCs and cancer cells within each well; the 384‐well format allowed the coverage of a vast library of chemicals simultaneously. The platform could monitor the growth phenotypes of cancer cells by imaging and biochemical readouts through cell proliferation and viability assessment. The authors tested over 2000 bioactive compounds on the HTiP and identified three potential antagonists that enhanced immune activity.^[^ ^163^ ^]^

Immune cells can respond to immunotherapeutic agents and biochemical cues in the TME with distinct functions, and these phenomena are represented by multiplexed phenotypic changes.^[^ ^164^ ^]^ Therefore, high‐throughput screening with a large number of cells for identification of certain phenotypes is crucial. The requirement for large sample sizes often increases operational and time costs that can be remedied by advanced screening approaches. Mair et al. designed a microfluidic chip system capable of performing genome‐wide loss‐of‐function screening of 10^8^ cells within one hour.^[^ ^82^ ^]^ Genome‐wide screening was achieved through CRISPR/Cas9 and cell sorting was achieved by immunomagnetically labeling the cells with magnetic particles coupled to antibodies. Under the magnetic guidance, cells with altered levels (low, medium, and high) of CD47 expression could fall into different chambers. CD47 on tumor cells function as a “do not eat me signal” that inhibits phagocytosis. The authors demonstrated identification of several genetic regulators of CD47, which could function as a prognostic biomarkers for checkpoint inhibitor immunotherapy.^[^ ^165^ ^]^ In order to reveal functional heterogeneity of cellular immunity on a single cell level, Ma et al. designed a microfluidic‐based chip for high‐content screening of the functional heterogeneity of immune cells. The chip comprised 1034 microchambers, each with 3 nL volume, to hold single cells for antibody‐encoded barcoding of phenotypes.^[^ ^86^ ^]^ Applicability of the chip system was demonstrated to distinguish antigen‐specific T cells between tumor patients and healthy donors by detecting perforin, interferon, and interleukin production. Polyfunctional T cells with higher cytokine production capabilities are indicated to be associated with successful vaccination,^[^ ^166^ ^]^ which potentially enables the platform as a predictor for the efficacy of a cancer vaccination.

Based on natural recognition between antigen‐specific T cells and peptide‐MHC complexes, efficient identification of antigen‐specific T cells can be achieved by high‐throughput production of peptide‐MHC complexes (pMHCs) as well. Stability of MHC molecules can be improved by introducing a disulfide bond without sacrificing its peptide binding ability. Two studies by Saini et al. and Moritz et al. utilized disulfide‐stabilized (DS) MHC I molecules for the purpose of screening cancer immunotherapy.^[^ ^167^, ^168^ ^]^ The DS approach and rapid one‐step loading of specific peptides enabled the production of vast libraries of peptides for pMHCs and facilitated the screening of neoantigen‐specific T cells while identifying off‐target risks from newly designed TCRs.^[^ ^169^ ^]^ As a high‐content format becomes a necessity for clinical and commercial use, new screening and engineering approaches should be developed with scalability in mind. Methods involving sequencing and CRISPR have long been used to generate enormous databases, while data regarding organoids has lagged behind due to nonstandardized analytical methods and sample restrictions. Bioprinting can be optimized to generate screening samples, such as spheroids, in parallel with low variability and high efficiency. Intricately designed organs‐on‐a‐chip systems may be challenging to adapt for high‐throughput screening because of fabrication and operation costs. However, microfluidic systems can facilitate single cell sorting for high‐throughput screening and drug administration combined with well‐plate system.

### 4.2 Artificial Intelligence

The capability to run assays in high‐content formats has contributed to the enormous increase in the amount of data collected from patients and biological samples. The enormous size of datasets has made manual evaluation cumbersome and ineffective. Additionally, manually analyzing data to find specific correlations is laborious and is likely to miss trends that are nonobvious or of interest. While the primary purpose of screening may be to assist in new drug discovery and diagnosis/treatment of an individual, the collective analysis of data from sets of patients can have an even greater value. Observing the trends and correlations found in these aggregate datasets can lead to a better understanding of complex diseases and contribute to the prevention and treatment of future occurrences.^[^ ^170^ ^]^ Therefore, machine learning methods are being implemented to draw valuable and actionable conclusions from otherwise impractical collections of data. These methods can be further segmented into supervised machine learning, which relies upon labeled datasets to make predictions based on past experience, unsupervised learning, which attempts to identify patterns and trends from previous occurrences, and reinforcement learning, which maximizes reward through making a sequential set of decisions.^[^ ^171^ ^]^ In each case, these algorithms require a training dataset to inform an analytical model.^[^ ^172^ ^]^ Historically, the training datasets have been from national initiatives such as TCGA; however, new training datasets can come from the other screening approaches mentioned earlier. Armed with the unique data derived from an array of screening approaches, AI can play an integral role in the pipeline of screening cancer immunotherapies, including predicting targetable epitopes for cancer vaccinations, pairing appropriate immunotherapeutics to responsive patients, and identifying adverse reactions prior to administration.

Recently, machine learning has been implemented in the identification of neoantigens presented by solid tumors. While the unique proteins produced by cancer cells may contain hundreds or thousands of amino acids, the MHC molecule only presents a segment of 9–25 peptides.^[^ ^173^ ^]^ Peptide presentation is further complicated by the high allelic variation in the MHC molecules presenting peptides due to polymorphism in the HLA genes responsible for MHC production.^[^ ^174^ ^]^ Biologically, this modularity and elevated degree of variation ensures that a larger variety of peptide sequences can be presented; however, it poses a significant challenge for those developing machine learning techniques to predict presentation. The ability to preemptively identify peptide sequences that bind well to MHC molecules can be used to accelerate the development of cancer vaccines and targeted therapies focusing on the neoantigens created by a tumor. Two studies have been recently published regarding unique algorithms for the prediction of these neoepitopes. While these papers have been extensively discussed by Moore and Nishimura,^[^ ^173^ ^]^ we will briefly discuss their broader impact on the development of immunotherapies. In both cases, studies trained their machine learning models (MixMHC2pred^[^ ^175^ ^]^ and MARIA,^[^ ^176^ ^]^ Racle et al. and Chen et al., respectively) on mass‐spectrometry‐derived peptidome datasets. The peptidome dataset used to train MixMHC2pred was roughly twice as large as MARIA (≈100 000 compared to ≈50 000 peptides) and encompassed a more diverse set of samples. Given a peptide sequence and the HLA allele, the MixMHC2pred program would present an MHC binding score as a metric of peptide‐MHC affinity. As a result, the prediction method showed an increase in true positives (MHCII‐presented peptides correctly predicted by the algorithm) relative to previous methods like NetMHCIIpan. MARIA, however, differs in its ability to factor in tissue‐specific gene expression to not simply predict the expression of a peptide, but its likelihood to elicit a strong T‐cell response. This supervised multimodal neural network also showed improvement in predictive capacity relative to models trained on isolated datasets of gene expression and mass spectrometry. With algorithms that can successfully predict the expression of specific peptide sequences by a tissue, cancer vaccines can be tailored to specific patients by better understanding the molecules required to elicit an immune response.

**Figure 7:**
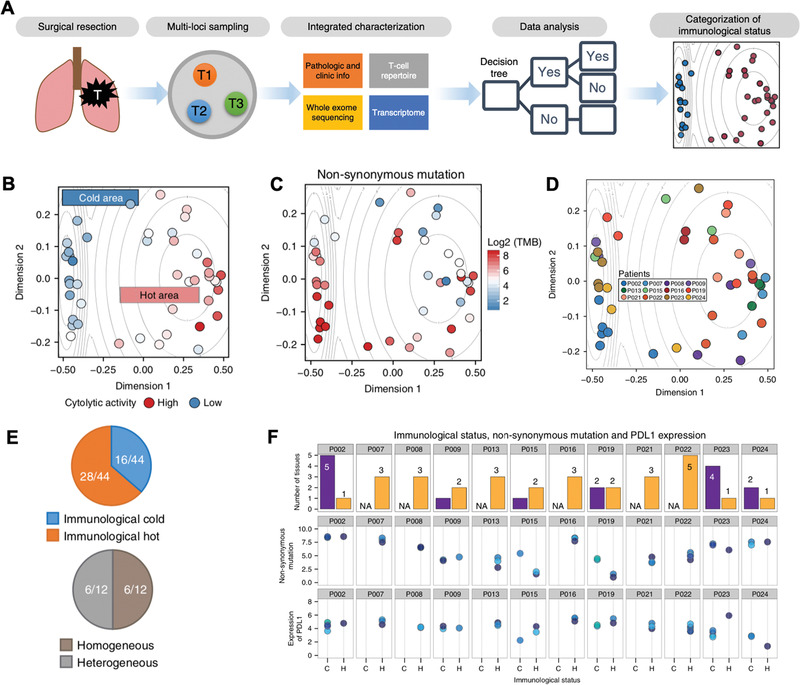
Machine learning for screening cancer immunotherapy. A) Schematic of applying machine learning on sequencing data gained from surgical biopsies of NSCLC patients. B) 2D visualization of an “immune map” showing immunologically “hot” (right) and “cold” (left) area from machine learning of multidimensional matrix of the input variables from NSCLC patients. Color indicates cytolytic activity. C) TMB was represented in the “immune map.” D) Location in the “immune map” of samples from different NSCLC patients represented as different colors. E) Pie chart (upper) displays proportion of immunologically “hot” and “cold” samples from all analyzed samples. Pie chart (lower) displays proportion of patients with different immunological status (heterogenous) and with one immunological status (homogenous). F) Immunological status, TMB, and PD‐L1 expression is categorized for each patient from top to bottom panel. The color in the middle and lower panel represent each individual patient. Reproduced with permission.^[^ ^69^ ^]^ Copyright 2018, Nature Publishing Group.

Massive databases of genomic and transcriptomic features derived from NGS have been compiled and algorithms can be trained on datasets containing patient characteristics and clinical outcomes. These analytical methods are highly diverse based upon the biomarkers that are studied and the patient populations from which the data has been drawn. As a National Cancer Institute (NCI) initiative between 2006 and 2018, TCGA amassed 2.5 petabytes of data characterizing the molecular characteristics of tumors. Using the established database, Choy et al. used an unsupervised shallow neural network to identify 16 new novel genes that were previously not believed to be related to the expression of immune checkpoint inhibitor molecules.^[^ ^68^ ^]^ Furthermore, analysis of over 13 000 samples parameterized into a 50‐dimensional space allowed the unsupervised network to identify an additional 18 genes that are correlated with the response of patients to immune checkpoint blockade.^[^ ^177^ ^]^ Though these genes are not yet reliable biomarkers indicative of patient response to treatment, further validation of algorithms like these can lead to new treatment paradigms and personalized medicine. Another study expanded the scope of the data collected within the TCGA to develop a database related to the immune landscape surrounding a tumor. The Cancer Immunome Atlas (TCIA) comprises characteristic information derived from the TCGA and immune checkpoint blockade clinical trials regarding the immune cells that have infiltrated 20 different types of solid tumors.^[^ ^178^ ^]^ To identify biomarkers of tumor immunity, the group implemented a random forest machine learning architecture that considered 127 different parameters and identified 26 features having a high impact on response to therapy. Based on the selected features associated with antigen presentation, checkpoint activation, and the presence of effector and suppressor cells, an immunophenoscore could be assigned. Further study demonstrated that the immunophenoscore could be used to reliably stratify patient groups into responders and nonresponders to immunotherapy while dramatically outperforming other singular biomarkers including PD1 expression and cytosolic activity. Facilitated by machine learning enabled data mining, Jia et al. utilized a random forest algorithm to generate an “immune map” of tumor loci based on multidimensional variables, such as neoantigen load, T‐cell repertoire, and crucial genes in regulating immune reactions and antigen presentation from WES, RNA‐seq, and T cell repertoire sequencing (**Figure** **7**). They examined spatial heterogeneity of the TME from different locations of tumor biopsies from one patient with NSCLC. By Gaussian maximum fitting, an “immune map” was represented as 2D with density contours indicating immunologically “hot” and “cold” regions. It was discovered that the TME is diverse within tumors and immunosuppressive mechanisms are heterogeneously presented. Because of this, single locus screening approaches may be insufficient for accurate response prediction and tumor characterization.^[^ ^69^ ^]^

Although well‐developed traditional approaches, such as flow cytometry, and various imaging techniques have already demonstrated their feasibility in tandem with machine learning, the advanced screening platforms discussed in this article can improve the accuracy, efficiency, and comprehensiveness of prognostic predictions. Developed algorithms can be readily applied to more advanced screening platforms. One machine learning approach, called FAUST, was developed to assist in the identification of unique cell populations in blood based on labeled cells observed with flow cytometry.^[^ ^179^ ^]^ The algorithm builds annotation trees by generating gating criteria for cells being observed; if the gating criteria that the algorithm sets produce a single population of cells, that gating strategy is no longer used. The result of this is the isolation of important features that differentiate cells from one another. While this study did not specifically tie features to response to immunotherapy, other work has shown that the presence, absence, and number of particular cell types derived from patient blood can be used to predict immunotherapeutic outcomes.^[^ ^180^ ^]^ Studies by Trebeschi et al. have already shown promise by analyzing the location, size, and morphology of tumors. Their random forest was able to select a singular radiomic biomarker based on a training set of 133 patient scans and was validated using an additional 70.^[^ ^181^ ^]^ While the success of the algorithm was highly dependent on the type of tumor being treated, the accuracy of the prediction can be greatly improved by expanding the dataset. Sun et al. took a similar radiomic approach in another study to train an algorithm to identify the extent of immune cell infiltration by creating 84 parameters to describe computed tomography (CT) scans.^[^ ^182^ ^]^ After identifying 8 essential radiomic features indicative of CD8 cell infiltration, a radiomic score was determined. This score was then validated against two additional datasets. When validated, it was determined that high radiomic scores correlated with improved overall patient survival. Though radiomics‐based approaches are minimally invasive and the imaging modalities are already essential components of the current standard of care, the generation of standardized datasets and the failure to directly assay cellular behavior in tumors may limit the broad scale adoption of these techniques.^[^ ^183^ ^]^ The AI algorithms developed for medical imaging analysis are expected to be adapted to the advanced screening models as well. Organ‐on‐a‐chip and bioprinting have different specialties in recreating complex cancer–immune crosstalk as mentioned earlier. Massive databases of analytical images and/or videos could be generated from these platforms to monitor cell systems in new ways to determine efficacy and drug–microscale tissue interactions. When scaling up sample diversity (for instance, patient samples and long‐term analysis), AI is expected to derive conclusions via algorithms similar to what is used in medical images analysis. Furthermore, artificial intelligence can be used to validate in vitro screening solutions by comparing the results from engineered screening models with in vivo or human trial data.

In short, machine learning and AI have the potential to revolutionize the clinical decision‐making processes surrounding the administration of immunotherapies. If the diversity of data input can be further enhanced with advanced screening approaches, patients can greatly benefit from the data‐driven approaches to cancer treatment.

## 5 Conclusions and Future Perspectives

In summary, the screening of cancer immunotherapies holds promise to surmount the many existing challenges preventing broad‐scale use in larger patient populations, especially in the identification of novel targets/mechanisms and predicting patient‐specific response. Various innovations across engineering disciplines have contributed to the creation of models with different focuses and specialties aiming to understand or recapitulate aspects of the tumor–immune interactions. To further expand the use of these platforms, they must be readily scaled up to rapid, high‐content modalities to meet the sample volumes and time constraints imposed by clinical use. Furthermore, screening model establishment and downstream data analysis should take cost, operational complexity, and physiological relevance into consideration. The implementation of AI in data analysis, specifically for cancer immunotherapies, shows promise to expedite screening pipelines by complementing engineering approaches. With surging numbers of clinical trials applying AI algorithms, high prediction accuracy and faithful alignment with clinical results is the one crucial golden standard in justifying its applicability and functionality.

However, future work is needed to address various challenges for screening cancer immunotherapies. The primary challenge involves the complexity of the tumor–immune‐therapeutic relationship and extraordinary heterogeneity in patient tumors. Most screening approaches try to emulate or characterize this complexity and heterogeneity but each has its own limitations. For example, even though loss‐of‐function screening via CRISPR/Cas platforms provides a large library of cellular models with unprecedented genetic heterogeneity, the mutational landscape of cancer cells cannot be exactly replicated through gene deletion alone. Additional modification with CRISPR can create gain‐of‐function models to enhance the relevance of the cell lines created for genomic^[^ ^184^ ^]^ and transcriptional regulation screening.^[^ ^185^ ^]^ Furthermore, different subsets of immune cells play distinct, crucial functions in determining the final treatment efficacy, but most in vitro platforms relying on organoids, bioprinting, and organs‐on‐a‐chip technologies have few, if any of these cell types. Novel derivation and fabrication strategies for tumor organoid engineering are promising as they provide a better representative sample of the immune population, while bioprinting and organs‐on‐a‐chip technologies more readily provide macroscopic spatiotemporal recapitulation of these interactions. The application of AI in immunotherapy development and screening is rapidly advancing, yet is still nascent for the screening approaches discussed due to the lack of standardized datasets. However, the various algorithms that have been developed can be meaningful references to reduce false‐positive and false‐negative interpretations.^[^ ^173^ ^]^ Some arguments have also been raised regarding bias in training sets and irreproducibility of certain algorithms for predicting response.^[^ ^186^ ^]^ The generalization of these algorithms to larger patient populations and the incorporation of patient outcomes with existing datasets will be crucial in advancing the power of these analytical methods.

Furthermore, engineering approaches designed for screening also need to be more compatible with the trend of clinical applications. Clinical results have indicated that an initial response to therapy does not guarantee sustained efficacy. Acquired resistance to certain treatment strategies, especially those acquired over time, is another challenge to be addressed in the screening methods.^[^ ^91^, ^187^ ^]^ Recent studies also suggest that the therapeutic efficacy of immunotherapies could be enhanced if used in tandem with the right combinations of adjuvant treatments.^[^ ^188^, ^189^, ^190^, ^191^ ^]^ Sparked by the FDA approval of nivolumab and ipilimumab as a combination therapy for metastatic melanoma, a surging number of trials are ongoing to test a variety of combination therapies.^[^ ^192^ ^]^ However, improved survival rates may also be accompanied by severe toxicity and extremely high cost.^[^ ^193^ ^]^ Clinical efficacy must justify the necessity for combination therapies and screening models can be an essential preclinical tool to maximize the number of combinations evaluated with holistic assessment of efficacy and safety. In addition, person‐to‐person variability is dramatic and identification of biomarkers for prediction is even more indispensable for combination immunotherapies due to their side effects and higher cost.^[^ ^194^ ^]^ In short, various challenges and clinically unmet needs in cancer immunotherapy are already being addressed by a variety of engineering approaches. In the future, not only will these systems become more refined to better model tumor behavior, but specific data analysis workflows facilitated by AI will improve our understanding of tumor–immune interactions and help improve the treatment of cancer.

## Conflict of Interest

S.S. owns equity in Akamara Therapeutics and Farcast Biosciences.

## References

[1] J. Couzin‐Frankel , Science 2010, 330, 440. 20966228 10.1126/science.330.6003.440

[2] I. Mellman , G. Coukos , G. Dranoff , Nature 2011, 480, 480. 22193102 10.1038/nature10673 PMC3967235

[3] F. S. Hodi , S. J. O'Day , D. F. McDermott , R. W. Weber , J. A. Sosman , J. B. Haanen , R. Gonzalez , C. Robert , D. Schadendorf , J. C. Hassel , W. Akerley , A. J. M. van den Eertwegh , J. Lutzky , P. Lorigan , J. M. Vaubel , G. P. Linette , D. Hogg , C. H. Ottensmeier , C. Lebbé , C. Peschel , I. Quirt , J. I. Clark , J. D. Wolchok , J. S. Weber , J. Tian , M. J. Yellin , G. M. Nichol , A. Hoos , W. J. Urba , N. Engl. J. Med. 2010, 363, 711. 20525992 10.1056/NEJMoa1003466 PMC3549297

[4] C. Robert , J. Schachter , G. V. Long , A. Arance , J. J. Grob , L. Mortier , A. Daud , M. S. Carlino , C. McNeil , M. Lotem , J. Larkin , P. Lorigan , B. Neyns , C. U. Blank , O. Hamid , C. Mateus , R. Shapira‐Frommer , M. Kosh , H. Zhou , N. Ibrahim , S. Ebbinghaus , A. Ribas , N. Engl. J. Med. 2015, 372, 2521. 25891173 10.1056/NEJMoa1503093

[5] E. B. Garon , N. A. Rizvi , R. Hui , N. Leighl , A. S. Balmanoukian , J. P. Eder , A. Patnaik , C. Aggarwal , M. Gubens , L. Horn , E. Carcereny , M.‐J. Ahn , E. Felip , J.‐S. Lee , M. D. Hellmann , O. Hamid , J. W. Goldman , J.‐C. Soria , M. Dolled‐Filhart , R. Z. Rutledge , J. Zhang , J. K. Lunceford , R. Rangwala , G. M. Lubiniecki , C. Roach , K. Emancipator , L. Gandhi , N. Engl. J. Med. 2015, 372, 2018. 25891174 10.1056/NEJMoa1501824

[6] J. Couzin‐Frankel , Science 2013, 342, 1432. 24357284 10.1126/science.342.6165.1432

[7] R. T. Manguso , H. W. Pope , M. D. Zimmer , F. D. Brown , K. B. Yates , B. C. Miller , N. B. Collins , K. Bi , M. W. LaFleur , V. R. Juneja , S. A. Weiss , J. Lo , D. E. Fisher , D. Miao , E. Van Allen , D. E. Root , A. H. Sharpe , J. G. Doench , W. N. Haining , Nature 2017, 547, 413. 28723893 10.1038/nature23270 PMC5924693

[8] S. L. Topalian , F. S. Hodi , J. R. Brahmer , S. N. Gettinger , D. C. Smith , D. F. McDermott , J. D. Powderly , R. D. Carvajal , J. A. Sosman , M. B. Atkins , P. D. Leming , D. R. Spigel , S. J. Antonia , L. Horn , C. G. Drake , D. M. Pardoll , L. Chen , W. H. Sharfman , R. A. Anders , J. M. Taube , T. L. McMiller , H. Xu , A. J. Korman , M. Jure‐Kunkel , S. Agrawal , D. McDonald , G. D. Kollia , A. Gupta , J. M. Wigginton , M. Sznol , N. Engl. J. Med. 2012, 366, 2443. 22658127 10.1056/NEJMoa1200690 PMC3544539

[9] M. S. Goldberg , Nat. Rev. Cancer 2019, 19, 587. 31492927 10.1038/s41568-019-0186-9

[10] D. J. Irvine , E. L. Dane , Nat. Rev. Immunol. 2020, 20, 321. 32005979 10.1038/s41577-019-0269-6 PMC7536618

[11] R. S. Riley , C. H. June , R. Langer , M. J. Mitchell , Nat. Rev. Drug Discovery 2019, 18, 175. 30622344 10.1038/s41573-018-0006-z PMC6410566

[12] P. Kvistborg , J. W. Yewdell , Science 2018, 359, 516. 29420276 10.1126/science.aar6574

[13] N. McGranahan , R. Rosenthal , C. T. Hiley , A. J. Rowan , T. B. Watkins , G. A. Wilson , N. J. Birkbak , S. Veeriah , P. Van Loo , J. Herrero , Cell 2017, 171, 1259.29107330

[14] E. Strønen , M. Toebes , S. Kelderman , M. M. van Buuren , W. Yang , N. van Rooij , M. Donia , M.‐L. Böschen , F. Lund‐Johansen , J. Olweus , T. N. Schumacher , Science 2016, 352, 1337. 27198675 10.1126/science.aaf2288

[15] J. G. Moffat , J. Rudolph , D. Bailey , Nat. Rev. Drug Discovery 2014, 13, 588. 25033736 10.1038/nrd4366

[16] H. Dong , S. E. Strome , D. R. Salomao , H. Tamura , F. Hirano , D. B. Flies , P. C. Roche , J. Lu , G. Zhu , K. Tamada , Nat. Med. 2002, 8, 793. 12091876 10.1038/nm730

[17] R. A. Morgan , M. E. Dudley , J. R. Wunderlich , M. S. Hughes , J. C. Yang , R. M. Sherry , R. E. Royal , S. L. Topalian , U. S. Kammula , N. P. Restifo , Science 2006, 314, 126. 16946036 10.1126/science.1129003 PMC2267026

[18] P. F. Robbins , R. A. Morgan , S. A. Feldman , J. C. Yang , R. M. Sherry , M. E. Dudley , J. R. Wunderlich , A. V. Nahvi , L. J. Helman , C. L. Mackall , J. Clin. Oncol. 2011, 29, 917. 21282551 10.1200/JCO.2010.32.2537 PMC3068063

[19] L. Scheetz , K. S. Park , Q. Li , P. R. Lowenstein , M. G. Castro , A. Schwendeman , J. J. Moon , Nat. Biomed. Eng. 2019, 3, 768. 31406259 10.1038/s41551-019-0436-x PMC6783331

[20] W. Sun , J. Lee , S. Zhang , C. Benyshek , M. R. Dokmeci , A. Khademhosseini , Adv. Sci. 2019, 6, 1801039. 10.1002/advs.201801039 PMC6325626 30643715

[21] R. Kuai , L. J. Ochyl , K. S. Bahjat , A. Schwendeman , J. J. Moon , Nat. Mater. 2017, 16, 489. 28024156 10.1038/nmat4822 PMC5374005

[22] U. Sahin , E. Derhovanessian , M. Miller , B.‐P. Kloke , P. Simon , M. Löwer , V. Bukur , A. D. Tadmor , U. Luxemburger , B. Schrörs , Nature 2017, 547, 222. 28678784 10.1038/nature23003

[23] U. Sahin , Ö. Türeci , Science 2018, 359, 1355. 29567706 10.1126/science.aar7112

[24] P. A. Ott , Z. Hu , D. B. Keskin , S. A. Shukla , J. Sun , D. J. Bozym , W. Zhang , A. Luoma , A. Giobbie‐Hurder , L. Peter , C. Chen , O. Olive , T. A. Carter , S. Li , D. J. Lieb , T. Eisenhaure , E. Gjini , J. Stevens , W. J. Lane , I. Javeri , K. Nellaiappan , A. M. Salazar , H. Daley , M. Seaman , E. I. Buchbinder , C. H. Yoon , M. Harden , N. Lennon , S. Gabriel , S. J. Rodig , D. H. Barouch , J. C. Aster , G. Getz , K. Wucherpfennig , D. Neuberg , J. Ritz , E. S. Lander , E. F. Fritsch , N. Hacohen , C. J. Wu , Nature 2017, 547, 217. 28678778 10.1038/nature22991 PMC5577644

[25] R. Macarron , M. N. Banks , D. Bojanic , D. J. Burns , D. A. Cirovic , T. Garyantes , D. V. S. Green , R. P. Hertzberg , W. P. Janzen , J. W. Paslay , U. Schopfer , G. S. Sittampalam , Nat. Rev. Drug Discovery 2011, 10, 188. 21358738 10.1038/nrd3368

[26] K.‐H. Yu , A. L. Beam , I. S. Kohane , Nat. Biomed. Eng. 2018, 2, 719. 31015651 10.1038/s41551-018-0305-z

[27] R. K. Harrison , Nat. Rev. Drug Discovery 2016, 15, 817. 27811931 10.1038/nrd.2016.184

[28] L. L. Siu , S. P. Ivy , E. L. Dixon , A. E. Gravell , S. A. Reeves , G. L. Rosner , Clin. Cancer Res. 2017, 23, 4950. 28864723 10.1158/1078-0432.CCR-16-3079 PMC5669041

[29] Y. Yang , J. Clin. Invest. 2015, 125, 3335. 26325031 10.1172/JCI83871 PMC4588312

[30] J. Lohmueller , O. J. Finn , Pharmacol. Ther. 2017, 178, 31. 28322974 10.1016/j.pharmthera.2017.03.008 PMC5600680

[31] D. M. Pardoll , Nat. Rev. Cancer 2012, 12, 252. 22437870 10.1038/nrc3239 PMC4856023

[32] W. Zou , L. Chen , Nat. Rev. Immunol. 2008, 8, 467. 18500231 10.1038/nri2326

[33] A. Ribas , J. D. Wolchok , Science 2018, 359, 1350. 29567705 10.1126/science.aar4060 PMC7391259

[34] E. S. Kim , J. E. Kim , M. A. Patel , A. Mangraviti , J. Ruzevick , M. Lim , J. Immunol. Res. 2016, 2016, 4683607. 26881264 10.1155/2016/4683607 PMC4736366

[35] H. O. Alsaab , S. Sau , R. Alzhrani , K. Tatiparti , K. Bhise , S. K. Kashaw , A. K. Iyer , Front. Pharmacol. 2017, 8, 561. 28878676 10.3389/fphar.2017.00561 PMC5572324

[36] C. Granier , E. De Guillebon , C. Blanc , H. Roussel , C. Badoual , E. Colin , A. Saldmann , A. Gey , S. Oudard , E. Tartour , ESMO Open 2017, 2, e000213. 28761757 10.1136/esmoopen-2017-000213 PMC5518304

[37] R. W. Jenkins , D. A. Barbie , K. T. Flaherty , Br. J. Cancer 2018, 118, 9. 29319049 10.1038/bjc.2017.434 PMC5765236

[38] S. L. Topalian , F. S. Hodi , J. R. Brahmer , S. N. Gettinger , D. C. Smith , D. F. McDermott , J. D. Powderly , R. D. Carvajal , J. A. Sosman , M. B. Atkins , N. Engl. J. Med. 2012, 366, 2443. 22658127 10.1056/NEJMoa1200690 PMC3544539

[39] M. S. Rooney , S. A. Shukla , C. J. Wu , G. Getz , N. Hacohen , Cell 2015, 160, 48. 25594174 10.1016/j.cell.2014.12.033 PMC4856474

[40] S. A. Rosenberg , J. Immunol. 2014, 192, 5451. 24907378 10.4049/jimmunol.1490019 PMC6293462

[41] T. Jiang , C. Zhou , S. Ren , OncoImmunology 2016, 5, e1163462. 27471638 10.1080/2162402X.2016.1163462 PMC4938354

[42] S. Lee , K. Margolin , Cancers 2011, 3, 3856. 24213115 10.3390/cancers3043856 PMC3763400

[43] M. Uhl , S. Aulwurm , J. Wischhusen , M. Weiler , J. Y. Ma , R. Almirez , R. Mangadu , Y.‐W. Liu , M. Platten , U. Herrlinger , Cancer Res. 2004, 64, 7954. 15520202 10.1158/0008-5472.CAN-04-1013

[44] H. Chi , C. Li , F. Zhao , L. Zhang , T. Ng , G. Jin , O. Sha , Front. Pharmacol. 2017, 8, 304. 28620298 10.3389/fphar.2017.00304 PMC5450331

[45] J. Fu , D. B. Kanne , M. Leong , L. H. Glickman , S. M. McWhirter , E. Lemmens , K. Mechette , J. J. Leong , P. Lauer , W. Liu , K. E. Sivick , Q. Zeng , K. C. Soares , L. Zheng , D. A. Portnoy , J. J. Woodward , D. M. Pardoll , T. W. Dubensky , Y. Kim , Sci. Transl. Med. 2015, 7, 283ra52. 10.1126/scitranslmed.aaa4306 PMC4504692 25877890

[46] S. A. Rosenberg , M. T. Lotze , L. M. Muul , A. E. Chang , F. P. Avis , S. Leitman , W. M. Linehan , C. N. Robertson , R. E. Lee , J. T. Rubin , C. A. Seipp , C. G. Simpson , D. E. White , N. Engl. J. Med. 1987, 316, 889. 3493432 10.1056/NEJM198704093161501

[47] S. A. Rosenberg , N. P. Restifo , Science 2015, 348, 62. 25838374 10.1126/science.aaa4967 PMC6295668

[48] S. A. Rosenberg , B. S. Packard , P. M. Aebersold , D. Solomon , S. L. Topalian , S. T. Toy , P. Simon , M. T. Lotze , J. C. Yang , C. A. Seipp , N. Engl. J. Med. 1988, 319, 1676. 3264384 10.1056/NEJM198812223192527

[49] A. D. Fesnak , C. H. June , B. L. Levine , Nat. Rev. Cancer 2016, 16, 566. 27550819 10.1038/nrc.2016.97 PMC5543811

[50] W. A. Lim , C. H. June , Cell 2017, 168, 724. 28187291 10.1016/j.cell.2017.01.016 PMC5553442

[51] M. L. Davila , R. J. Brentjens , Clin. Adv. Hematol. Oncol. 2016, 14, 802. 27930631 PMC5536094

[52] M. J. Frigault , J. Lee , M. C. Basil , C. Carpenito , S. Motohashi , J. Scholler , O. U. Kawalekar , S. Guedan , S. E. McGettigan , A. D. Posey , S. Ang , L. J. N. Cooper , J. M. Platt , F. B. Johnson , C. M. Paulos , Y. Zhao , M. Kalos , M. C. Milone , C. H. June , Cancer Immunol. Res. 2015, 3, 356. 25600436 10.1158/2326-6066.CIR-14-0186 PMC4390458

[53] S. S. Neelapu , F. L. Locke , N. L. Bartlett , L. J. Lekakis , D. B. Miklos , C. A. Jacobson , I. Braunschweig , O. O. Oluwole , T. Siddiqi , Y. Lin , N. Engl. J. Med. 2017, 377, 2531. 29226797 10.1056/NEJMoa1707447 PMC5882485

[54] S. L. Maude , T. W. Laetsch , J. Buechner , S. Rives , M. Boyer , H. Bittencourt , P. Bader , M. R. Verneris , H. E. Stefanski , G. D. Myers , N. Engl. J. Med. 2018, 378, 439. 29385370 10.1056/NEJMoa1709866 PMC5996391

[55] C. Linnemann , B. Heemskerk , P. Kvistborg , R. J. C. Kluin , D. A. Bolotin , X. Chen , K. Bresser , M. Nieuwland , R. Schotte , S. Michels , R. Gomez‐Eerland , L. Jahn , P. Hombrink , N. Legrand , C. J. Shu , I. Z. Mamedov , A. Velds , C. U. Blank , J. B. A. G. Haanen , M. A. Turchaninova , R. M. Kerkhoven , H. Spits , S. R. Hadrup , M. H. M. Heemskerk , T. Blankenstein , D. M. Chudakov , G. M. Bendle , T. N. M. Schumacher , Nat. Med. 2013, 19, 1534. 24121928 10.1038/nm.3359

[56] J. C. Fitzgerald , S. L. Weiss , S. L. Maude , D. M. Barrett , S. F. Lacey , J. J. Melenhorst , P. Shaw , R. A. Berg , C. H. June , D. L. Porter , N. V. Frey , S. A. Grupp , D. T. Teachey , Crit. Care Med. 2017, 45, e124. 27632680 10.1097/CCM.0000000000002053 PMC5452983

[57] S. Rafiq , C. S. Hackett , R. J. Brentjens , Nat. Rev. Clin. Oncol. 2019, 17, 147. 31848460 10.1038/s41571-019-0297-y PMC7223338

[58] R. E. Hollingsworth , K. Jansen , npj Vaccines 2019, 4, 7. 30774998 10.1038/s41541-019-0103-y PMC6368616

[59] C. Guo , M. H. Manjili , J. R. Subjeck , D. Sarkar , P. B. Fisher , X.‐Y. Wang , Adv. Cancer Res. 2013, 119, 421. 23870514 10.1016/B978-0-12-407190-2.00007-1 PMC3721379

[60] C. L.‐L. Chiang , G. Coukos , L. E. Kandalaft , Vaccines 2015, 3, 344. 26343191 10.3390/vaccines3020344 PMC4494356

[61] E. C. Hsueh , R. Essner , L. J. Foshag , D. W. Ollila , G. Gammon , S. J. O'Day , P. D. Boasberg , S. L. Stern , X. Ye , D. L. Morton , J. Clin. Oncol. 2002, 20, 4549. 12454111 10.1200/JCO.2002.01.151

[62] P. W. Kantoff , C. S. Higano , N. D. Shore , E. R. Berger , E. J. Small , D. F. Penson , C. H. Redfern , A. C. Ferrari , R. Dreicer , R. B. Sims , Y. Xu , M. W. Frohlich , P. F. Schellhammer , N. Engl. J. Med. 2010, 363, 411. 20818862 10.1056/NEJMoa1001294

[63] A. D. Garg , P. G. Coulie , B. J. Van den Eynde , P. Agostinis , Trends Immunol. 2017, 38, 577. 28610825 10.1016/j.it.2017.05.006

[64] N. Pardi , M. J. Hogan , F. W. Porter , D. Weissman , Nat. Rev. Drug Discovery 2018, 17, 261. 29326426 10.1038/nrd.2017.243 PMC5906799

[65] T. N. Schumacher , R. D. Schreiber , Science 2015, 348, 69. 25838375 10.1126/science.aaa4971

[66] L. Li , S. P. Goedegebuure , W. E. Gillanders , Ann. Oncol. 2017, 28, xii11. 29253113 10.1093/annonc/mdx681 PMC5834106

[67] E. M. Van Allen , D. Miao , B. Schilling , S. A. Shukla , C. Blank , L. Zimmer , A. Sucker , U. Hillen , M. H. Geukes Foppen , S. M. Goldinger , J. Utikal , J. C. Hassel , B. Weide , K. C. Kaehler , C. Loquai , P. Mohr , R. Gutzmer , R. Dummer , S. Gabriel , C. J. Wu , D. Schadendorf , L. A. Garraway , Science 2015, 350, 207. 26359337 10.1126/science.aad0095 PMC5054517

[68] C. T. Choy , C. H. Wong , S. L. Chan , Front. Genet. 2019, 9, 682. 30662451 10.3389/fgene.2018.00682 PMC6329279

[69] Q. Jia , W. Wu , Y. Wang , P. B. Alexander , C. Sun , Z. Gong , J.‐N. Cheng , H. Sun , Y. Guan , X. Xia , L. Yang , X. Yi , Y. Y. Wan , H. Wang , J. He , P. A. Futreal , Q.‐J. Li , B. Zhu , Nat. Commun. 2018, 9, 5361. 30560866 10.1038/s41467-018-07767-w PMC6299138

[70] C. Zheng , L. Zheng , J.‐K. Yoo , H. Guo , Y. Zhang , X. Guo , B. Kang , R. Hu , J. Y. Huang , Q. Zhang , Z. Liu , M. Dong , X. Hu , W. Ouyang , J. Peng , Z. Zhang , Cell 2017, 169, 1342. 28622514 10.1016/j.cell.2017.05.035

[71] M. Yadav , S. Jhunjhunwala , Q. T. Phung , P. Lupardus , J. Tanguay , S. Bumbaca , C. Franci , T. K. Cheung , J. Fritsche , T. Weinschenk , Z. Modrusan , I. Mellman , J. R. Lill , L. Delamarre , Nature 2014, 515, 572. 25428506 10.1038/nature14001

[72] S. J. Patel , N. E. Sanjana , R. J. Kishton , A. Eidizadeh , S. K. Vodnala , M. Cam , J. J. Gartner , L. Jia , S. M. Steinberg , T. N. Yamamoto , A. S. Merchant , G. U. Mehta , A. Chichura , O. Shalem , E. Tran , R. Eil , M. Sukumar , E. P. Guijarro , C.‐P. Day , P. Robbins , S. Feldman , G. Merlino , F. Zhang , N. P. Restifo , Nature 2017, 548, 537. 28783722 10.1038/nature23477 PMC5870757

[73] D. Pan , A. Kobayashi , P. Jiang , L. Ferrari de Andrade , R. E. Tay , A. M. Luoma , D. Tsoucas , X. Qiu , K. Lim , P. Rao , H. W. Long , G.‐C. Yuan , J. Doench , M. Brown , X. S. Liu , K. W. Wucherpfennig , Science 2018, 359, 770. 29301958 10.1126/science.aao1710 PMC5953516

[74] E. Shifrut , J. Carnevale , V. Tobin , T. L. Roth , J. M. Woo , C. T. Bui , P. J. Li , M. E. Diolaiti , A. Ashworth , A. Marson , Cell 2018, 175, 1958. 30449619 10.1016/j.cell.2018.10.024 PMC6689405

[75] J. T. Neal , X. Li , J. Zhu , V. Giangarra , C. L. Grzeskowiak , J. Ju , I. H. Liu , S.‐H. Chiou , A. A. Salahudeen , A. R. Smith , B. C. Deutsch , L. Liao , A. J. Zemek , F. Zhao , K. Karlsson , L. M. Schultz , T. J. Metzner , L. D. Nadauld , Y.‐Y. Tseng , S. Alkhairy , C. Oh , P. Keskula , D. Mendoza‐Villanueva , F. M. De La Vega , P. L. Kunz , J. C. Liao , J. T. Leppert , J. B. Sunwoo , C. Sabatti , J. S. Boehm , W. C. Hahn , G. X. Y. Zheng , M. M. Davis , C. J. Kuo , Cell 2018, 175, 1972. 30550791 10.1016/j.cell.2018.11.021 PMC6656687

[76] K. I. Votanopoulos , S. Forsythe , H. Sivakumar , A. Mazzocchi , J. Aleman , L. Miller , E. Levine , P. Triozzi , A. Skardal , Ann. Surg. Oncol. 2019, 27, 1956. 31858299 10.1245/s10434-019-08143-8 PMC7474462

[77] K. K. Dijkstra , C. M. Cattaneo , F. Weeber , M. Chalabi , J. van de Haar , L. F. Fanchi , M. Slagter , D. L. van der Velden , S. Kaing , S. Kelderman , N. van Rooij , M. E. van Leerdam , A. Depla , E. F. Smit , K. J. Hartemink , R. de Groot , M. C. Wolkers , N. Sachs , P. Snaebjornsson , K. Monkhorst , J. Haanen , H. Clevers , T. N. Schumacher , E. E. Voest , Cell 2018, 174, 1586. 30100188 10.1016/j.cell.2018.07.009 PMC6558289

[78] F. Jacob , R. D. Salinas , D. Y. Zhang , P. T. T. Nguyen , J. G. Schnoll , S. Z. H. Wong , R. Thokala , S. Sheikh , D. Saxena , S. Prokop , D.‐a. Liu , X. Qian , D. Petrov , T. Lucas , H. I. Chen , J. F. Dorsey , K. M. Christian , Z. A. Binder , M. Nasrallah , S. Brem , D. M. O'Rourke , G.‐l. Ming , H. Song , Cell 2020, 180, 188. 31883794 10.1016/j.cell.2019.11.036 PMC7556703

[79] M. A. Heinrich , R. Bansal , T. Lammers , Y. S. Zhang , R. Michel Schiffelers , J. Prakash , Adv. Mater. 2019, 31, 1806590. 10.1002/adma.201806590 30702785

[80] S. J. Turley , V. Cremasco , J. L. Astarita , Nat. Rev. Immunol. 2015, 15, 669. 26471778 10.1038/nri3902

[81] R. W. Jenkins , A. R. Aref , P. H. Lizotte , E. Ivanova , S. Stinson , C. W. Zhou , M. Bowden , J. Deng , H. Liu , D. Miao , M. X. He , W. Walker , G. Zhang , T. Tian , C. Cheng , Z. Wei , S. Palakurthi , M. Bittinger , H. Vitzthum , J. W. Kim , A. Merlino , M. Quinn , C. Venkataramani , J. A. Kaplan , A. Portell , P. C. Gokhale , B. Phillips , A. Smart , A. Rotem , R. E. Jones , L. Keogh , M. Anguiano , L. Stapleton , Z. Jia , M. Barzily‐Rokni , I. Cañadas , T. C. Thai , M. R. Hammond , R. Vlahos , E. S. Wang , H. Zhang , S. Li , G. J. Hanna , W. Huang , M. P. Hoang , A. Piris , J.‐P. Eliane , A. O. Stemmer‐Rachamimov , L. Cameron , M.‐J. Su , P. Shah , B. Izar , M. Thakuria , N. R. LeBoeuf , G. Rabinowits , V. Gunda , S. Parangi , J. M. Cleary , B. C. Miller , S. Kitajima , R. Thummalapalli , B. Miao , T. U. Barbie , V. Sivathanu , J. Wong , W. G. Richards , R. Bueno , C. H. Yoon , J. Miret , M. Herlyn , L. A. Garraway , E. M. Van Allen , G. J. Freeman , P. T. Kirschmeier , J. H. Lorch , P. A. Ott , F. S. Hodi , K. T. Flaherty , R. D. Kamm , G. M. Boland , K.‐K. Wong , D. Dornan , C. P. Paweletz , D. A. Barbie , Cancer Discovery 2018, 8, 196. 29101162 10.1158/2159-8290.CD-17-0833 PMC5809290

[82] B. Mair , P. M. Aldridge , R. S. Atwal , D. Philpott , M. Zhang , S. N. Masud , M. Labib , A. H. Y. Tong , E. H. Sargent , S. Angers , J. Moffat , S. O. Kelley , Nat. Biomed. Eng. 2019, 3, 796. 31548591 10.1038/s41551-019-0454-8

[83] D. Park , K. Son , Y. Hwang , J. Ko , Y. Lee , J. Doh , N. L. Jeon , Front. Immunol. 2019, 10, 1133. 31191524 10.3389/fimmu.2019.01133 PMC6546835

[84] A. I. Segaliny , G. Li , L. Kong , C. Ren , X. Chen , J. K. Wang , D. Baltimore , G. Wu , W. Zhao , Lab Chip 2018, 18, 3733. 30397689 10.1039/c8lc00818c PMC6279597

[85] L. Y. Ke , Z. K. Kuo , Y. S. Chen , T. Y. Yeh , M. Dong , H. W. Tseng , C. H. Liu , Lab Chip 2017, 18, 106. 29211085 10.1039/c7lc00963a

[86] C. Ma , R. Fan , H. Ahmad , Q. Shi , B. Comin‐Anduix , T. Chodon , R. C. Koya , C.‐C. Liu , G. A. Kwong , C. G. Radu , A. Ribas , J. R. Heath , Nat. Med. 2011, 17, 738. 21602800 10.1038/nm.2375 PMC3681612

[87] A. A. Friedman , A. Letai , D. E. Fisher , K. T. Flaherty , Nat. Rev. Cancer 2015, 15, 747. 26536825 10.1038/nrc4015 PMC4970460

[88] P. S. Hegde , D. S. Chen , Immunity 2020, 52, 17. 31940268 10.1016/j.immuni.2019.12.011

[89] M. S. Lawrence , P. Stojanov , P. Polak , G. V. Kryukov , K. Cibulskis , A. Sivachenko , S. L. Carter , C. Stewart , C. H. Mermel , S. A. Roberts , Nature 2013, 499, 214. 23770567 10.1038/nature12213 PMC3919509

[90] R. S. Herbst , J.‐C. Soria , M. Kowanetz , G. D. Fine , O. Hamid , M. S. Gordon , J. A. Sosman , D. F. McDermott , J. D. Powderly , S. N. Gettinger , H. E. K. Kohrt , L. Horn , D. P. Lawrence , S. Rost , M. Leabman , Y. Xiao , A. Mokatrin , H. Koeppen , P. S. Hegde , I. Mellman , D. S. Chen , F. S. Hodi , Nature 2014, 515, 563. 25428504 10.1038/nature14011 PMC4836193

[91] P. Sharma , S. Hu‐Lieskovan , J. A. Wargo , A. Ribas , Cell 2017, 168, 707. 28187290 10.1016/j.cell.2017.01.017 PMC5391692

[92] J. Kondo , M. Inoue , Cells 2019, 8, 470. 10.3390/cells8050470 PMC6562517 31108870

[93] X. Ma , J. Liu , W. Zhu , M. Tang , N. Lawrence , C. Yu , M. Gou , S. Chen , Adv. Drug Delivery Rev. 2018, 132, 235. 10.1016/j.addr.2018.06.011 PMC6226327 29935988

[94] K. L. Fetah , B. J. DiPardo , E.‐M. Kongadzem , J. S. Tomlinson , A. Elzagheid , M. Elmusrati , A. Khademhosseini , N. Ashammakhi , Small 2019, 15, 1901985. 10.1002/smll.201901985 PMC6929691 31724305

[95] A. K. Miri , E. Mostafavi , D. Khorsandi , S.‐K. Hu , M. Malpica , A. Khademhosseini , Biofabrication 2019, 11, 042002. 31170695 10.1088/1758-5090/ab2798 PMC6756175

[96] D. Chowell , L. G. Morris , C. M. Grigg , J. K. Weber , R. M. Samstein , V. Makarov , F. Kuo , S. M. Kendall , D. Requena , N. Riaz , Science 2018, 359, 582. 29217585 10.1126/science.aao4572 PMC6057471

[97] R. M. Samstein , C.‐H. Lee , A. N. Shoushtari , M. D. Hellmann , R. Shen , Y. Y. Janjigian , D. A. Barron , A. Zehir , E. J. Jordan , A. Omuro , Nat. Genet. 2019, 51, 202. 30643254 10.1038/s41588-018-0312-8 PMC6365097

[98] D. Miao , C. A. Margolis , N. I. Vokes , D. Liu , A. Taylor‐Weiner , S. M. Wankowicz , D. Adeegbe , D. Keliher , B. Schilling , A. Tracy , Nat. Genet. 2018, 50, 1271. 30150660 10.1038/s41588-018-0200-2 PMC6119118

[99] J. E. Rosenberg , J. Hoffman‐Censits , T. Powles , M. S. Van Der Heijden , A. V. Balar , A. Necchi , N. Dawson , P. H. O'Donnell , A. Balmanoukian , Y. Loriot , Lancet 2016, 387, 1909. 26952546 10.1016/S0140-6736(16)00561-4 PMC5480242

[100] S. Goodwin , J. D. McPherson , W. R. McCombie , Nat. Rev. Genet. 2016, 17, 333. 27184599 10.1038/nrg.2016.49 PMC10373632

[101] K. Schwarze , J. Buchanan , J. C. Taylor , S. Wordsworth , Genet. Med. 2018, 20, 1122. 29446766 10.1038/gim.2017.247

[102] P. J. Park , Nat. Rev. Genet. 2009, 10, 669. 19736561 10.1038/nrg2641 PMC3191340

[103] W. Hugo , H. Shi , L. Sun , M. Piva , C. Song , X. Kong , G. Moriceau , A. Hong , K. B. Dahlman , D. B. Johnson , J. A. Sosman , A. Ribas , R. S. Lo , Cell 2015, 162, 1271. 26359985 10.1016/j.cell.2015.07.061 PMC4821508

[104] Z. Wang , M. Gerstein , M. Snyder , Nat. Rev. Genet. 2009, 10, 57. 19015660 10.1038/nrg2484 PMC2949280

[105] D. P. Carbone , M. Reck , L. Paz‐Ares , B. Creelan , L. Horn , M. Steins , E. Felip , M. M. van den Heuvel , T.‐E. Ciuleanu , F. Badin , N. Engl. J. Med. 2017, 376, 2415. 28636851 10.1056/NEJMoa1613493 PMC6487310

[106] S. A. Gujar , D. Pan , P. Marcato , K. A. Garant , P. W. K. Lee , Mol. Ther. 2011, 19, 797. 21245852 10.1038/mt.2010.297 PMC3070098

[107] A. Han , J. Glanville , L. Hansmann , M. M. Davis , Nat. Biotechnol. 2014, 32, 684. 24952902 10.1038/nbt.2938 PMC4337815

[108] B. Wiedenheft , S. H. Sternberg , J. A. Doudna , Nature 2012, 482, 331. 22337052 10.1038/nature10886

[109] L. Cong , F. A. Ran , D. Cox , S. Lin , R. Barretto , N. Habib , P. D. Hsu , X. Wu , W. Jiang , L. A. Marraffini , F. Zhang , Science 2013, 339, 819. 23287718 10.1126/science.1231143 PMC3795411

[110] P. Mali , L. Yang , K. M. Esvelt , J. Aach , M. Guell , J. E. DiCarlo , J. E. Norville , G. M. Church , Science 2013, 339, 823. 23287722 10.1126/science.1232033 PMC3712628

[111] J. Luo , Trends Cancer 2016, 2, 313. 28603775 10.1016/j.trecan.2016.05.001 PMC5461962

[112] A. Agrotis , R. Ketteler , Front. Genet. 2015, 6, 300. 26442115 10.3389/fgene.2015.00300 PMC4585242

[113] M. S. Lawrence , P. Stojanov , P. Polak , G. V. Kryukov , K. Cibulskis , A. Sivachenko , S. L. Carter , C. Stewart , C. H. Mermel , S. A. Roberts , A. Kiezun , P. S. Hammerman , A. McKenna , Y. Drier , L. Zou , A. H. Ramos , T. J. Pugh , N. Stransky , E. Helman , J. Kim , C. Sougnez , L. Ambrogio , E. Nickerson , E. Shefler , M. L. Cortés , D. Auclair , G. Saksena , D. Voet , M. Noble , D. DiCara , P. Lin , L. Lichtenstein , D. I. Heiman , T. Fennell , M. Imielinski , B. Hernandez , E. Hodis , S. Baca , A. M. Dulak , J. Lohr , D.‐A. Landau , C. J. Wu , J. Melendez‐Zajgla , A. Hidalgo‐Miranda , A. Koren , S. A. McCarroll , J. Mora , R. S. Lee , B. Crompton , R. Onofrio , M. Parkin , W. Winckler , K. Ardlie , S. B. Gabriel , C. W. M. Roberts , J. A. Biegel , K. Stegmaier , A. J. Bass , L. A. Garraway , M. Meyerson , T. R. Golub , D. A. Gordenin , S. Sunyaev , E. S. Lander , G. Getz , Nature 2013, 499, 214. 23770567 10.1038/nature12213 PMC3919509

[114] J. M. Zaretsky , A. Garcia‐Diaz , D. S. Shin , H. Escuin‐Ordinas , W. Hugo , S. Hu‐Lieskovan , D. Y. Torrejon , G. Abril‐Rodriguez , S. Sandoval , L. Barthly , J. Saco , B. Homet Moreno , R. Mezzadra , B. Chmielowski , K. Ruchalski , I. P. Shintaku , P. J. Sanchez , C. Puig‐Saus , G. Cherry , E. Seja , X. Kong , J. Pang , B. Berent‐Maoz , B. Comin‐Anduix , T. G. Graeber , P. C. Tumeh , T. N. M. Schumacher , R. S. Lo , A. Ribas , N. Engl. J. Med. 2016, 375, 819. 27433843 10.1056/NEJMoa1604958 PMC5007206

[115] T. A. Chan , J. D. Wolchok , A. Snyder , N. Engl. J. Med. 2015, 373, 1984. 26559592 10.1056/NEJMc1508163

[116] N. A. Rizvi , M. D. Hellmann , A. Snyder , P. Kvistborg , V. Makarov , J. J. Havel , W. Lee , J. Yuan , P. Wong , T. S. Ho , M. L. Miller , N. Rekhtman , A. L. Moreira , F. Ibrahim , C. Bruggeman , B. Gasmi , R. Zappasodi , Y. Maeda , C. Sander , E. B. Garon , T. Merghoub , J. D. Wolchok , T. N. Schumacher , T. A. Chan , Science 2015, 348, 124. 25765070 10.1126/science.aaa1348 PMC4993154

[117] E. Tran , M. Ahmadzadeh , Y.‐C. Lu , A. Gros , S. Turcotte , P. F. Robbins , J. J. Gartner , Z. Zheng , Y. F. Li , S. Ray , J. R. Wunderlich , R. P. Somerville , S. A. Rosenberg , Science 2015, 350, 1387. 26516200 10.1126/science.aad1253 PMC7445892

[118] J. Ren , X. Liu , C. Fang , S. Jiang , C. H. June , Y. Zhao , Clin. Cancer Res. 2017, 23, 2255. 27815355 10.1158/1078-0432.CCR-16-1300 PMC5413401

[119] O. Parnas , M. Jovanovic , T. M. Eisenhaure , R. H. Herbst , A. Dixit , C. J. Ye , D. Przybylski , R. J. Platt , I. Tirosh , N. E. Sanjana , O. Shalem , R. Satija , R. Raychowdhury , P. Mertins , S. A. Carr , F. Zhang , N. Hacohen , A. Regev , Cell 2015, 162, 675. 26189680 10.1016/j.cell.2015.06.059 PMC4522370

[120] M. W. LaFleur , T. H. Nguyen , M. A. Coxe , K. B. Yates , J. D. Trombley , S. A. Weiss , F. D. Brown , J. E. Gillis , D. J. Coxe , J. G. Doench , W. N. Haining , A. H. Sharpe , Nat. Commun. 2019, 10, 1668. 30971695 10.1038/s41467-019-09656-2 PMC6458184

[121] F. Weeber , M. van de Wetering , M. Hoogstraat , K. K. Dijkstra , O. Krijgsman , T. Kuilman , C. G. M. Gadellaa‐van Hooijdonk , D. L. van der Velden , D. S. Peeper , E. P. J. G. Cuppen , R. G. Vries , H. Clevers , E. E. Voest , Proc. Natl. Acad. Sci. USA 2015, 112, 13308. 26460009 10.1073/pnas.1516689112 PMC4629330

[122] J. Drost , H. Clevers , Nat. Rev. Cancer 2018, 18, 407. 29692415 10.1038/s41568-018-0007-6

[123] D. Tuveson , H. Clevers , Science 2019, 364, 952. 31171691 10.1126/science.aaw6985

[124] A. Mullard , Nat. Rev. Drug Discovery 2018, 17, 613. 10.1038/nrd.2018.154 30160254

[125] N. Sachs , J. de Ligt , O. Kopper , E. Gogola , G. Bounova , F. Weeber , A. V. Balgobind , K. Wind , A. Gracanin , H. Begthel , J. Korving , R. van Boxtel , A. A. Duarte , D. Lelieveld , A. van Hoeck , R. F. Ernst , F. Blokzijl , I. J. Nijman , M. Hoogstraat , M. van de Ven , D. A. Egan , V. Zinzalla , J. Moll , S. F. Boj , E. E. Voest , L. Wessels , P. J. van Diest , S. Rottenberg , R. G. J. Vries , E. Cuppen , H. Clevers , Cell 2018, 172, 373. 29224780 10.1016/j.cell.2017.11.010

[126] D. Gao , I. Vela , A. Sboner , P. J. Iaquinta , W. R. Karthaus , A. Gopalan , C. Dowling , J. N. Wanjala , E. A. Undvall , V. K. Arora , J. Wongvipat , M. Kossai , S. Ramazanoglu , L. P. Barboza , W. Di , Z. Cao , Q. F. Zhang , I. Sirota , L. Ran , T. Y. MacDonald , H. Beltran , J.‐M. Mosquera , K. A. Touijer , P. T. Scardino , V. P. Laudone , K. R. Curtis , D. E. Rathkopf , M. J. Morris , D. C. Danila , S. F. Slovin , S. B. Solomon , J. A. Eastham , P. Chi , B. Carver , M. A. Rubin , H. I. Scher , H. Clevers , C. L. Sawyers , Y. Chen , Cell 2014, 159, 176. 25201530 10.1016/j.cell.2014.08.016 PMC4237931

[127] M. van de Wetering , H. E. Francies , J. M. Francis , G. Bounova , F. Iorio , A. Pronk , W. van Houdt , J. van Gorp , A. Taylor‐Weiner , L. Kester , Cell 2015, 161, 933. 25957691 10.1016/j.cell.2015.03.053 PMC6428276

[128] S. F. Boj , C.‐I. Hwang , L. A. Baker , I. I. C. Chio , D. Engle , V. Corbo , M. Jager , M. Ponz‐Sarvise , H. Tiriac , M. S. Spector , A. Gracanin , T. Oni , K. H. Yu , R. van Boxtel , M. Huch , K. D. Rivera , J. P. Wilson , M. E. Feigin , D. Öhlund , A. Handly‐Santana , C. M. Ardito‐Abraham , M. Ludwig , E. Elyada , B. Alagesan , G. Biffi , G. N. Yordanov , B. Delcuze , B. Creighton , K. Wright , Y. Park , F. H. M. Morsink , I. Q. Molenaar , I. H. B. Rinkes , E. Cuppen , Y. Hao , Y. Jin , I. J. Nijman , C. Iacobuzio‐Donahue , S. D. Leach , D. l J. Pappin , M. Hammell , D. S. Klimstra , O. Basturk , R. H. Hruban , G. J. Offerhaus , R. G. J. Vries , H. Clevers , D. A. Tuveson , Cell 2015, 160, 324. 25557080 10.1016/j.cell.2014.12.021 PMC4334572

[129] L. Broutier , G. Mastrogiovanni , M. M. A. Verstegen , H. E. Francies , L. M. Gavarró , C. R. Bradshaw , G. E. Allen , R. Arnes‐Benito , O. Sidorova , M. P. Gaspersz , N. Georgakopoulos , B.‐K. Koo , S. Dietmann , S. E. Davies , R. K. Praseedom , R. Lieshout , J. N. M. Ijzermans , S. J. Wigmore , K. Saeb‐Parsy , M. J. Garnett , L. J. W. van der Laan , M. Huch , Nat. Med. 2017, 23, 1424. 29131160 10.1038/nm.4438 PMC5722201

[130] M. E. Dudley , J. R. Wunderlich , T. E. Shelton , J. Even , S. A. Rosenberg , J. Immunother. 2003, 26, 332. 12843795 10.1097/00002371-200307000-00005 PMC2305721

[131] W. Liu , Y. S. Zhang , M. A. Heinrich , F. De Ferrari , H. L. Jang , S. M. Bakht , M. M. Alvarez , J. Yang , Y. C. Li , G. Trujillo‐de Santiago , Adv. Mater. 2017, 29, 1604630. 10.1002/adma.201604630 PMC5235978 27859710

[132] Y. S. Zhang , M. Duchamp , R. Oklu , L. W. Ellisen , R. Langer , A. Khademhosseini , ACS Biomater. Sci. Eng. 2016, 2, 1710. 28251176 10.1021/acsbiomaterials.6b00246 PMC5328669

[133] C. Colosi , S. R. Shin , V. Manoharan , S. Massa , M. Costantini , A. Barbetta , M. R. Dokmeci , M. Dentini , A. Khademhosseini , Adv. Mater. 2016, 28, 677. 26606883 10.1002/adma.201503310 PMC4804470

[134] Y. S. Zhang , K. Yue , J. Aleman , K. Mollazadeh‐Moghaddam , S. M. Bakht , J. Yang , W. Jia , V. Dell'Erba , P. Assawes , S. R. Shin , Ann. Biomed. Eng. 2017, 45, 148. 27126775 10.1007/s10439-016-1612-8 PMC5085899

[135] P. S. Gungor‐Ozkerim , I. Inci , Y. S. Zhang , A. Khademhosseini , M. R. Dokmeci , Biomater. Sci. 2018, 6, 915. 29492503 10.1039/c7bm00765e PMC6439477

[136] Y. Zhao , R. Yao , L. Ouyang , H. Ding , T. Zhang , K. Zhang , S. Cheng , W. Sun , Biofabrication 2014, 6, 035001. 24722236 10.1088/1758-5082/6/3/035001

[137] T. Q. Huang , X. Qu , J. Liu , S. Chen , Biomed. Microdevices 2014, 16, 127. 24150602 10.1007/s10544-013-9812-6 PMC3945947

[138] W. Peng , D. Unutmaz , I. T. Ozbolat , Trends Biotechnol. 2016, 34, 722. 27296078 10.1016/j.tibtech.2016.05.013

[139] M. H. Zaman , L. M. Trapani , A. L. Sieminski , D. MacKellar , H. Gong , R. D. Kamm , A. Wells , D. A. Lauffenburger , P. Matsudaira , Proc. Natl. Acad. Sci. USA 2006, 103, 10889. 16832052 10.1073/pnas.0604460103 PMC1544144

[140] M. Huse , Nat. Rev. Immunol. 2017, 17, 679. 28757604 10.1038/nri.2017.74 PMC6312705

[141] B. Grigoryan , S. J. Paulsen , D. C. Corbett , D. W. Sazer , C. L. Fortin , A. J. Zaita , P. T. Greenfield , N. J. Calafat , J. P. Gounley , A. H. Ta , F. Johansson , A. Randles , J. E. Rosenkrantz , J. D. Louis‐Rosenberg , P. A. Galie , K. R. Stevens , J. S. Miller , Science 2019, 364, 458. 31048486 10.1126/science.aav9750 PMC7769170

[142] L. E. Bertassoni , M. Cecconi , V. Manoharan , M. Nikkhah , J. Hjortnaes , A. L. Cristino , G. Barabaschi , D. Demarchi , M. R. Dokmeci , Y. Yang , A. Khademhosseini , Lab Chip 2014, 14, 2202. 24860845 10.1039/c4lc00030g PMC4201051

[143] W. Jia , P. S. Gungor‐Ozkerim , Y. S. Zhang , K. Yue , K. Zhu , W. Liu , Q. Pi , B. Byambaa , M. R. Dokmeci , S. R. Shin , A. Khademhosseini , Biomaterials 2016, 106, 58. 27552316 10.1016/j.biomaterials.2016.07.038 PMC5300870

[144] J. S. Miller , K. R. Stevens , M. T. Yang , B. M. Baker , D.‐H. T. Nguyen , D. M. Cohen , E. Toro , A. A. Chen , P. A. Galie , X. Yu , Nat. Mater. 2012, 11, 768. 22751181 10.1038/nmat3357 PMC3586565

[145] D. F. Quail , R. L. Bowman , L. Akkari , M. L. Quick , A. J. Schuhmacher , J. T. Huse , E. C. Holland , J. C. Sutton , J. A. Joyce , Science 2016, 352, aad3018. 27199435 10.1126/science.aad3018 PMC5450629

[146] J. M. Grolman , D. Zhang , A. M. Smith , J. S. Moore , K. A. Kilian , Adv. Mater. 2015, 27, 5512. 26283579 10.1002/adma.201501729 PMC4745120

[147] X. Cao , R. Ashfaq , F. Cheng , S. Maharjan , J. Li , G. Ying , S. Hassan , H. Xiao , K. Yue , Y. S. Zhang , Adv. Funct. Mater. 2019, 29, 1807173. 10.1002/adfm.201807173 PMC7546431 33041741

[148] B. Zhang , A. Korolj , B. F. L. Lai , M. Radisic , Nat. Rev. Mater. 2018, 3, 257.

[149] S. N. Bhatia , D. E. Ingber , Nat. Biotechnol. 2014, 32, 760. 25093883 10.1038/nbt.2989

[150] G. Adriani , A. Pavesi , A. T. Tan , A. Bertoletti , J. P. Thiery , R. D. Kamm , Drug Discovery Today 2016, 21, 1472. 27185084 10.1016/j.drudis.2016.05.006 PMC5035566

[151] a) A. Polini , L. Loretta , A. Barra , Y. S. Zhang , F. Calabi , G. Gigli , Drug Discovery Today 2019, 24, 517; 30312743 10.1016/j.drudis.2018.10.003 PMC6440212

[152] S. E. Park , A. Georgescu , D. Huh , Science 2019, 364, 960. 31171693 10.1126/science.aaw7894 PMC7764943

[153] T. Takebe , B. Zhang , M. Radisic , Cell Stem Cell 2017, 21, 297. 28886364 10.1016/j.stem.2017.08.016

[154] Y. S. Zhang , J. Aleman , S. R. Shin , T. Kilic , D. Kim , S. A. Mousavi Shaegh , S. Massa , R. Riahi , S. Chae , N. Hu , H. Avci , W. Zhang , A. Silvestri , A. Sanati Nezhad , A. Manbohi , F. De Ferrari , A. Polini , G. Calzone , N. Shaikh , P. Alerasool , E. Budina , J. Kang , N. Bhise , J. Ribas , A. Pourmand , A. Skardal , T. Shupe , C. E. Bishop , M. R. Dokmeci , A. Atala , A. Khademhosseini , Proc. Natl. Acad. Sci. USA 2017, 114, E2293. 28265064 10.1073/pnas.1612906114 PMC5373350

[155] V. S. Shirure , Y. Bi , M. B. Curtis , A. Lezia , M. M. Goedegebuure , S. P. Goedegebuure , R. Aft , R. C. Fields , S. C. George , Lab Chip 2018, 18, 3687. 30393802 10.1039/c8lc00596f PMC10644986

[156] S. Nagrath , L. V. Sequist , S. Maheswaran , D. W. Bell , D. Irimia , L. Ulkus , M. R. Smith , E. L. Kwak , S. Digumarthy , A. Muzikansky , P. Ryan , U. J. Balis , R. G. Tompkins , D. A. Haber , M. Toner , Nature 2007, 450, 1235. 18097410 10.1038/nature06385 PMC3090667

[157] G. Schwank , B.‐K. Koo , V. Sasselli , J. F. Dekkers , I. Heo , T. Demircan , N. Sasaki , S. Boymans , E. Cuppen , C. K. van der Ent , Cell Stem Cell 2013, 13, 653. 24315439 10.1016/j.stem.2013.11.002

[158] M. Matano , S. Date , M. Shimokawa , A. Takano , M. Fujii , Y. Ohta , T. Watanabe , T. Kanai , T. Sato , Nat. Med. 2015, 21, 256. 25706875 10.1038/nm.3802

[159] E. Driehuis , H. Clevers , Am. J. Physiol.: Gastrointest. Liver Physiol. 2017, 312, G257. 28126704 10.1152/ajpgi.00410.2016

[160] K. Fetah , P. Tebon , M. J. Goudie , J. Eichenbaum , L. Ren , N. Barros , R. Nasiri , S. Ahadian , N. Ashammakhi , M. R. Dokmeci , A. Khademhosseini , Prog. Biomed. Eng. 2019, 1, 012001.

[161] F. Meng , C. M. Meyer , D. Joung , D. A. Vallera , M. C. McAlpine , A. Panoskaltsis‐Mortari , Adv. Mater. 2019, 31, 1806899. 10.1002/adma.201806899 PMC6996245 30663123

[162] N. Phan , J. J. Hong , B. Tofig , M. Mapua , D. Elashoff , N. A. Moatamed , J. Huang , S. Memarzadeh , R. Damoiseaux , A. Soragni , Commun. Biol. 2019, 2, 78. 30820473 10.1038/s42003-019-0305-x PMC6389967

[163] X. Mo , C. Tang , Q. Niu , T. Ma , Y. Du , H. Fu , Cell Chem. Biol. 2019, 26, 331. 30639259 10.1016/j.chembiol.2018.11.011 PMC6501824

[164] J. J. O'Shea , C. A. Hunter , R. N. Germain , Nat. Immunol. 2008, 9, 450. 18425095 10.1038/ni0508-450

[165] M. E. Logtenberg , J. M. Jansen , M. Raaben , M. Toebes , K. Franke , A. M. Brandsma , H. L. Matlung , A. Fauster , R. Gomez‐Eerland , N. A. Bakker , Nat. Med. 2019, 25, 612. 30833751 10.1038/s41591-019-0356-z PMC7025889

[166] P. A. Darrah , D. T. Patel , P. M. De Luca , R. W. Lindsay , D. F. Davey , B. J. Flynn , S. T. Hoff , P. Andersen , S. G. Reed , S. L. Morris , Nat. Med. 2007, 13, 843. 17558415 10.1038/nm1592

[167] S. K. Saini , T. Tamhane , R. Anjanappa , A. Saikia , S. Ramskov , M. Donia , I. M. Svane , S. N. Jakobsen , M. Garcia‐Alai , M. Zacharias , R. Meijers , S. Springer , S. R. Hadrup , Sci. Immunol. 2019, 4, eaau9039. 31324690 10.1126/sciimmunol.aau9039

[168] A. Moritz , R. Anjanappa , C. Wagner , S. Bunk , M. Hofmann , G. Pszolla , A. Saikia , M. Garcia‐Alai , R. Meijers , H.‐G. Rammensee , S. Springer , D. Maurer , Sci. Immunol. 2019, 4, eaav0860. 31324691 10.1126/sciimmunol.aav0860

[169] W. Shao , P. G. Pedrioli , W. Wolski , C. Scurtescu , E. Schmid , J. A. Vizcaíno , M. Courcelles , H. Schuster , D. Kowalewski , F. Marino , Nucleic Acids Res. 2018, 46, D1237. 28985418 10.1093/nar/gkx664 PMC5753376

[170] A. Alyass , M. Turcotte , D. Meyre , BMC Med. Genomics 2015, 8, 33. 26112054 10.1186/s12920-015-0108-y PMC4482045

[171] a) M. van Otterlo , M. Wiering , in Reinforcement Learning: State‐of‐the‐Art (Eds: Wiering M., van Otterlo M.), Springer, Berlin 2012, p. 3;

[172] J. Wiens , E. S. Shenoy , Clin. Infect. Dis. 2018, 66, 149. 29020316 10.1093/cid/cix731 PMC5850539

[173] T. V. Moore , M. I. Nishimura , Nat. Rev. Clin. Oncol. 2019, 17, 71. 10.1038/s41571-019-0315-0 PMC7223749 31836878

[174] K. K. Jensen , M. Andreatta , P. Marcatili , S. Buus , J. A. Greenbaum , Z. Yan , A. Sette , B. Peters , M. Nielsen , Immunology 2018, 154, 394. 29315598 10.1111/imm.12889 PMC6002223

[175] J. Racle , J. Michaux , G. A. Rockinger , M. Arnaud , S. Bobisse , C. Chong , P. Guillaume , G. Coukos , A. Harari , C. Jandus , M. Bassani‐Sternberg , D. Gfeller , Nat. Biotechnol. 2019, 37, 1283. 31611696 10.1038/s41587-019-0289-6

[176] B. Chen , M. S. Khodadoust , N. Olsson , L. E. Wagar , E. Fast , C. L. Liu , Y. Muftuoglu , B. J. Sworder , M. Diehn , R. Levy , M. M. Davis , J. E. Elias , R. B. Altman , A. A. Alizadeh , Nat. Biotechnol. 2019, 37, 1332. 31611695 10.1038/s41587-019-0280-2 PMC7075463

[177] C. Choy , C. Wong , S. Chan , Ann. Oncol. 2018, 29, viii22.

[178] P. Charoentong , F. Finotello , M. Angelova , C. Mayer , M. Efremova , D. Rieder , H. Hackl , Z. Trajanoski , Cell Rep. 2017, 18, 248. 28052254 10.1016/j.celrep.2016.12.019

[179] E. Becht , M. Headley , E. Newell , R. Gottardo , J. Immunol. 2020, 204, 159.2.31748348

[180] C. Krieg , M. Nowicka , S. Guglietta , S. Schindler , F. J. Hartmann , L. M. Weber , R. Dummer , M. D. Robinson , M. P. Levesque , B. Becher , Nat. Med. 2018, 24, 144. 29309059 10.1038/nm.4466

[181] S. Trebeschi , S. G. Drago , N. J. Birkbak , I. Kurilova , A. M. Cǎlin , A. Delli Pizzi , F. Lalezari , D. M. J. Lambregts , M. W. Rohaan , C. Parmar , E. A. Rozeman , K. J. Hartemink , C. Swanton , J. B. A. G. Haanen , C. U. Blank , E. F. Smit , R. G. H. Beets‐Tan , H. J. W. L. Aerts , Ann. Oncol. 2019, 30, 998. 30895304 10.1093/annonc/mdz108 PMC6594459

[182] R. Sun , E. J. Limkin , M. Vakalopoulou , L. Dercle , S. Champiat , S. R. Han , L. Verlingue , D. Brandao , A. Lancia , S. Ammari , A. Hollebecque , J.‐Y. Scoazec , A. Marabelle , C. Massard , J.‐C. Soria , C. Robert , N. Paragios , E. Deutsch , C. Ferté , Lancet Oncol. 2018, 19, 1180. 30120041 10.1016/S1470-2045(18)30413-3

[183] E. Deutsch , N. Paragios , Ann. Oncol. 2019, 30, 879. 31124559 10.1093/annonc/mdz150

[184] T. S. Klann , J. B. Black , M. Chellappan , A. Safi , L. Song , I. B. Hilton , G. E. Crawford , T. E. Reddy , C. A. Gersbach , Nat. Biotechnol. 2017, 35, 561. 28369033 10.1038/nbt.3853 PMC5462860

[185] S. Konermann , M. D. Brigham , A. E. Trevino , J. Joung , O. O. Abudayyeh , C. Barcena , P. D. Hsu , N. Habib , J. S. Gootenberg , H. Nishimasu , O. Nureki , F. Zhang , Nature 2015, 517, 583. 25494202 10.1038/nature14136 PMC4420636

[186] J. A. Carter , P. Gilbo , G. S. Atwal , Nat. Med. 2019, 25, 1833. 31806907 10.1038/s41591-019-0671-4

[187] N. P. Restifo , M. J. Smyth , A. Snyder , Nat. Rev. Cancer 2016, 16, 121. 26822578 10.1038/nrc.2016.2 PMC6330026

[188] C. Schmidt , Nature 2017, 552, S67. 10.1038/d41586-017-08702-7 32094671

[189] C. G. Drake , Ann. Oncol. 2012, 23, viii41. 22918927 10.1093/annonc/mds262 PMC6278955

[190] D. N. Khalil , E. L. Smith , R. J. Brentjens , J. D. Wolchok , Nat. Rev. Clin. Oncol. 2016, 13, 273. 26977780 10.1038/nrclinonc.2016.25 PMC5551685

[191] J. Galon , D. Bruni , Nat. Rev. Drug Discovery 2019, 18, 197. 30610226 10.1038/s41573-018-0007-y

[192] J. S. Lopez , U. Banerji , Nat. Rev. Clin. Oncol. 2017, 14, 57. 27377132 10.1038/nrclinonc.2016.96 PMC6135233

[193] J. D. Wolchok , V. Chiarion‐Sileni , R. Gonzalez , P. Rutkowski , J.‐J. Grob , C. L. Cowey , C. D. Lao , J. Wagstaff , D. Schadendorf , P. F. Ferrucci , N. Engl. J. Med. 2017, 377, 1345. 28889792 10.1056/NEJMoa1709684 PMC5706778

[194] P. A. Ott , Z. Hu , D. B. Keskin , S. A. Shukla , J. Sun , D. J. Bozym , W. Zhang , A. Luoma , A. Giobbie‐Hurder , L. Peter , Nature 2017, 547, 217. 28678778 10.1038/nature22991 PMC5577644

